# Treatment Effects of Removable Functional Appliances in Pre-Pubertal and Pubertal Class II Patients: A Systematic Review and Meta-Analysis of Controlled Studies

**DOI:** 10.1371/journal.pone.0141198

**Published:** 2015-10-28

**Authors:** Giuseppe Perinetti, Jasmina Primožič, Lorenzo Franchi, Luca Contardo

**Affiliations:** 1 Department of Medical, Surgical and Health Sciences, University of Trieste, Trieste, Italy; 2 Department of Orthodontics and Jaw Orthopaedics, Medical Faculty, University of Ljubljana, Ljubljana, Slovenia; 3 Department of Orthodontics, School of Dentistry, University of Florence, Florence, Italy, and Department of Orthodontics and Pediatric Dentistry, School of Dentistry, The University of Michigan, Ann Arbor, Michigan, United States of America; University of North Carolina at Chapel Hill, UNITED STATES

## Abstract

**Background:**

Treatment effects of removable functional appliances in Class II malocclusion patients according to the pre-pubertal or pubertal growth phase has yet to be clarified.

**Objectives:**

To assess and compare skeletal and dentoalveolar effects of removable functional appliances in Class II malocclusion treatment between pre-pubertal and pubertal patients.

**Search methods:**

Literature survey using the Medline, SCOPUS, LILACS and SciELO databases, the Cochrane Library from inception to May 31, 2015. A manual search was also performed.

**Selection criteria:**

Randomised (RCTs) or controlled clinical trials with a matched untreated control group. No restrictions were set regarding the type of removable appliance whenever used alone.

**Data collection and analysis:**

For the meta-analysis, cephalometric parameters on the supplementary mandibular growth were the main outcomes, with other cephalometric parameters considered as secondary outcomes. Risk of bias in individual and across studies were evaluated along with sensitivity analysis for low quality studies. Mean differences and 95% confidence intervals for annualised changes were computed according to a random model. Differences between pre-pubertal and pubertal patients were assessed by subgroup analyses. GRADE assessment was performed for the main outcomes.

**Results:**

Twelve articles (but only 3 RCTs) were included accounting for 8 pre-pubertal and 7 pubertal groups. Overall supplementary total mandibular length and mandibular ramus height were 0.95 mm (0.38, 1.51) and 0.00 mm (-0.52, 0.53) for pre-pubertal patients and 2.91 mm (2.04, 3.79) and 2.18 mm (1.51, 2.86) for pubertal patients, respectively. The subgroup difference was significant for both parameters (p<0.001). No maxillary growth restrain or increase in facial divergence was seen in either subgroup. The GRADE assessment was low for the pre-pubertal patients, and generally moderate for the pubertal patients.

**Conclusions:**

Taking into account the limited quality and heterogeneity of the included studies, functional treatment by removable appliances may be effective in treating Class II malocclusion with clinically relevant skeletal effects if performed during the pubertal growth phase.

## Introduction

The mandibular condyles, including their cartilage, have a primary role in the development and growth of the oro-facial complex. In this regard, a deficient growth of the condyles may results in mandibular retrognathia, also referred as skeletal Class II malocclusion. Interestingly, animal studies have shown that forward mandibular displacement enhances condylar growth resulting in significant changes in the morphology of the Mandible [1], [2]. Such induced condylar growth has been shown to be characterized by a thickness of the condrogenic, proliferative, and hypertrophic layers of condylar cartilage on the posterior aspect of the condyle, thus yielding to an increase in total mandibular length [1], [2].

According to this biological evidence, an orthopaedic approach to treat skeletal Class II malocclusion in growing subjects is based on forward positioning of the mandible [3]. For this purpose, several removable or fixed appliances have been developed [3]. However, reviews reported very limited [4–6], partial [7] or relevant [8], [9] effectiveness of such treatment in terms of additional mandibular growth, i.e. correction of skeletal Class II malocclusion. The reason for this apparently inconsistent evidence might reside in the different interventions performed [8], [9] in the large variation in individual responsiveness to functional treatment [10], or in the timing, i.e. pre-pubertal or pubertal growth phase [11], during which treatment is performed. Indeed, growth does not occur at a constant rate and children of the same chronological age might not have equivalent skeletal maturity or growth potential [11]. Interestingly, while previous reviews focused mainly on the appliance type [7], [12], none has focused on the timing of intervention, although this issue has been raised years ago [8]. The only exception is a recent meta-analysis [13] on fixed appliances that reported significant skeletal effects for pubertal patients and not for post-pubertal ones.

A further ethical issue also relates to the clinical trials evaluating the effectiveness of functional treatment for skeletal Class II malocclusion. Indeed, leaving subjects with relevant malocclusions without orthodontic treatment during the pubertal growth phase or after, has limited the execution of randomized clinical trials (RCTs) at this stage of development. Therefore, reviews including exclusively RCTs [4], [5], might have been focused mostly on pre-pubertal subjects, leaving the potential effects of treatment on pubertal patients excluded from the analysis. For this reason, the consideration of controlled clinical trials (CCTs) with reasonable methodological quality has been advocated [14]. Moreover, it has been reported that whenever RCTs are not available for meta-analysis, CCTs or observational studies may be used with essentially similar outcomes [15].

Whether the efficiency of functional treatment for skeletal Class II malocclusion is critically dependent on the timing of intervention has still not been clarified, especially for removable appliances. Yet, this information would have relevant clinical implications in terms of treatment planning. Therefore, the aim of the present review and meta-analysis of RCTs and CCTs was to assess the short-term skeletal (mainly supplementary mandibular growth) and dentoalveolar effects of removable functional appliances for the treatment of Class II malocclusion during the pre-pubertal or pubertal growth phase, as compared to matched untreated controls.

## Materials and Methods

### Search strategy

The present meta-analysis followed the Preferred Reporting Items for Systematic Reviews and Meta-Analyses (PRISMA) statement [16] (S1 PRISMA Checklist), used a previous systematic review as a template [13], and it has been registered at the PROSPERO database (http://www.crd.york.ac.uk/PROSPERO). Articles were identified through a literature survey carried out through the following databases: i) PubMed, ii) SCOPUS iii) Latin American and Caribbean Health Sciences (LILACS), iv) Scientific Electronic Library Online (SciELO), and v) The Cochrane Library. The survey covered the period from inceptions to the last access on May 31, 2015 with no language restrictions. The search algorithms used in each database have been published previously [13] and are reported in Table 1. Finally, a manual search was also performed by scoring the references within the studies examined and the titles of the papers published over the last 15 years among the following major journals: i) American Journal of Orthodontics and Dentofacial Orthopedics, ii) European Journal of Orthodontics, iii) Journal of Orofacial Orthopedics, iv) Korean Journal of Orthodontics, v) Orthodontics and Craniofacial Research; vi) Progress in Orthodontics, vii) The Angle Orthodontist, and viii) World Journal of Orthodontics. The eligibility assessment was performed independently by two blinded authors (GP and JP). The intra-examiner reliability in the study selection process was assessed through the Cohen k test assuming a threshold value of 0.61 [17]. Conflicts were resolved by discussion of each article, until consensus was reached. An attempt to contact the corresponding Authors of the included studies was made to retrieve any missing information or clarification of specific items.

**Table 1:** The search algorithms used in the literature search according to each database.

Database	Algorithm	Hits
**Medline, Entrez PubMed** www.ncbi.nlm.nih.gov	"Orthodontic appliances, Functional"[Mesh] OR "Orthodontic appliances"[All Fields] OR "functional"[All Fields] AND ("Malocclusion, Angle Class II"[Mesh] OR "jaw"[All Fields] OR "orthop*"[All Fields]) AND (("Class"[All Fields] AND "II"[All Fields] AND "Malocclusion"[All Fields]) OR ("Angle"[All Fields] AND "Class"[All Fields] AND "II"[All Fields]))	2,087
**SCOPUS** www.scopus.com	TITLE-ABS-KEY(((orthodontic appliance) OR (functional jaw orthopedics)) AND ((class ii malocclusion) OR (angle class ii))) AND (LIMIT-TO(DOCTYPE, "ar") OR LIMIT-TO(DOCTYPE, "ip")) AND (LIMIT-TO(SUBJAREA, "DENT") OR LIMIT-TO(SUBJAREA, "MULT"))	1,303
**LILACS** http://lilacs.bvsalud.org	((Orthodontic appliance) OR (Functional jaw orthopedics)) AND ((Class II malocclusion) OR (Angle Class II))	251
**Cochrane Library (Registered Controlled trials)** www.thecochranelibrary.com	((Orthodontic appliance) OR (Functional jaw orthopedics)) AND ((Class II malocclusion) OR (Angle Class II))	215
**SciELO** http://www.scielo.org	((Orthodontic appliance) OR (Functional jaw orthopedics)) AND ((Class II malocclusion) OR (Angle Class II))	28

### Eligibility criteria

The studies retrieved had to be RCTs or either prospective or retrospective CCTs. They had to include healthy patients treated during the pre-pubertal or pubertal growth phases. These studies had to investigate the skeletal and dentoalveolar effects with no restriction as to the type of parameters collected, as long as at least one of the main outcomes (see below) was included. Also, no restrictions were set regarding the type of removable appliance whenever used alone without any other additional therapy (fixed, extra-oral traction, etc.), treatment length or to the cephalometric analysis used. Studies were excluded if a reliable indicator of growth phase (hand-and-wrist maturation [HWM] method or cervical vertebral maturation [CVM] method) was not used. Further inclusions and exclusion criteria are listed in detail in Table 2.

**Table 2:** Inclusion and exclusion criteria used in the present review.

**Inclusion criteria**
**1.** Longitudinal studies, either prospective or retrospective, on healthy growing subjects treated for skeletal Class II malocclusion due to mandibular retrusion
**2.** Use of removable functional orthodontic appliances
**3.** Use of a reliable indicator of individual skeletal maturity to assess treatment timing that had to be either pre-pubertal or pubertal
**4.** Use of matched control groups of untreated Class II malocclusion subjects with similar growth phase
**5.** Reporting treatment effects data according to parameters collected before and at the end of functional treatments
**Exclusion criteria**
**1.** Case reports, case series with no statistical analysis, comments, letters to the Editor, reviews
**2.** Studies using the headgear alone or in combination with other functional appliances
**3.** Studies in which the compared treated groups were subjected to different treatment modalities
**4.** Studies in which treatment length was significantly different than the observational time length of the control group
**5.** Studies in which orthodontic treatment was combined with fixed appliances, mini-implants or surgery
**6.** Studies without cephalometric analyses or without measures defined herein as primary outcomes
**7.** Studies in which a favourable response to treatment (according to the Authors’ definition) was an inclusion criterion
**8.** Studies in which skeletal maturation was assessed but subjects with different stages were pooled in the same treated or control group
**9.** Studies in which the control group was based on published reference standards without a specific matching of the groups by age, gender, and other features

### Data items

The following data were extracted independently by two authors (GP and JP): study design, prospective or retrospective enrolment of the treated group, sample size, gender distribution, age, type of functional appliance used, Class II description, indicators of skeletal maturity and distribution of subjects according to growth phase, prognostic or other features, cephalometric magnification factor, full treatment and observational duration, mandibular advancement for treated patients and when treatment was stopped. Regarding the treatment effects, the following items were also collected: success rate (as defined in different studies), skeletal, dentoalveolar and soft tissues effects, and Authors’ conclusions on the growth phase and treatment efficiency. Forms used for data extraction were mostly pre-defined at the protocol stage by two authors (GP and LC).

### Assessment of risk of bias in individual studies

No single approach in assessing methodological soundness may be appropriate to all systematic reviews [18]. Therefore, risk of bias in individual studies was assessed according to the Cochrane Collaboration’s Tool [19] and a slightly modified Downs and Black tool [20] for randomised and non-randomised trials, respectively.

The items included in the Cochrane Collaboration’s Tool [19] are defined as: sequence generation, allocation concealment, blinding, incomplete outcome data (i.e., drop-out information or cephalometric magnification), selective outcome reporting (i.e., relevant cephalometric parameters), and other risks of bias. In particular, the ‘other bias’ domain included a set of pre-specified entries defined as: i) inclusion of Class II patients relying on overjet alone, which cannot account for a true skeletal Class II malocclusion [21]; ii) lack of analysis of other potentially relevant diagnostic/prognostic features, such as facial divergence, maxillary protrusion, or condylar angle [10].

The original Downs and Black tool is calculated by rating each study across a variety of domains including reporting (10 items), external validity (3 items), internal validity—bias (7 items), internal validity—confounding (6 items), and power (1 item) with maximum score of 32 [20]. In the present review, only minor adaptations were followed to adhere with the studies dealing with functional treatment for Class II malocclusion. These were as follows: i) items were added in the reporting section as: ‘Were inclusion and exclusion criteria clearly stated?’ (yes, 1 point; no or unclear, 0 points); ‘Is the Class II malocclusion fully described?’ (fully described including skeletal parameters, or at least reporting a full molar Class II, 1 point; no, 0 points); ii) the original item #14 ‘Was an attempt made to blind study subjects to the intervention they have received?’ was removed as this is not applicable; iii) the original item #20 ‘Were the main outcome measures accurate (reliable and repeatable)?’ was used to derive 2 items for the reliability of the skeletal maturation staging and cephalometric measurements (yes, 1 point; no or unclear, 0 points); iii) The last item on the power was simplified as follows: ‘Prior estimate of sample size’ (yes, 1 point; no or unclear, 0 points). The maximum score for this modified Downs and Black tool is thus 29.Evaluation was performed without blinding by two Authors (GP and JP) and conflicts were resolved by discussion. A third Author (LC) was consulted if necessary.

### Assessment of risk of bias across studies

Heterogeneity was assessed using the χ2-based Q-statistic method with a significant p value <0.1. However, because of the moderate insensitivity of the Q statistic [22], an I^2^ index was also reported with values ≥50% considered associated to a substantial heterogeneity among the studies [23]. In particular, the I^2^ index describes the percentage of total variation across studies due to heterogeneity rather than chance. The tau^2^ was also calculated for the heterogeneity assessment. The Review Manager software version 5.2.6 (http://www.cochrane.org) was used for the assessment of heterogeneity. Moreover, the Egger test [24] and the Begg and Mazumdar rank correlation test [25] were employed to assess publication bias and to compensate for possible lack of power [26], with significant p value set at p<0.1. Calculations were performed using the Comprehensive Meta-Analysis software version 2.0 (Biostat Inc., Englewood, NJ, USA).

### Primary and secondary outcomes

For the meta-analysis, primary outcomes included those cephalometric parameters related to mandibular growth, and expressed as supplementary growth in comparison to the untreated controls. They were: 1) total mandibular length, 2) mandibular ramus height, 3) composite mandibular length (according to Pancherz Analysis) [27], and 4) mandibular base (according to Pancherz Analysis) [27]. Secondary outcomes, again as supplementary changes in comparison to the untreated controls, were: 1) SNA, 2) SNB and 3) ANB angles, 4) maxillary base (according to Pancherz Analysis) [27], 5) total facial divergence, and 6) mandibular incisor proclination (relative to the mandibular plane). Although the measures of total mandibular length, mandibular ramus height, facial divergence, and lower incisor proclination differed slightly among the studies, these were combined in the overall effects according to the concept that the differences in the intra-group changes would be poorly sensitive to the absolute measures from which they are derived.

### Summary measures and synthesis of results

The mean difference was used for statistical pooling of data and results were expressed as mean and 95% confidence intervals (CIs). Moreover, 90% prediction intervals were also calculated as previously reported [28]. Subgroup analyses were performed whenever possible according to the growth phase, pubertal or post-pubertal, during which treatment was performed. Moreover, to account for the heterogeneity of the treatments, i.e. differences among the appliance used, treatment length, and cephalometric analysis, a random effect model was used for calculations of all the overall effects [29]. No studies including two or more treated groups compared to a single control group were retrieved. Finally, these analyses were reported according to the different subgroups of pre-pubertal and pubertal subjects and shown through forest plots. Treatment duration was noteworthy different among the retrieved studies; therefore, when not already reported in the articles, annualised changes for all the parameters were calculated and used for meta-analysis. Furthermore, whenever necessary and possible, the magnification for linear parameters was set at 0%. The Review Manager software was used for meta-analysis (S1 Table).

### Additional analysis

As for the main analyses, all the additional analyses were performed according to the pre-pubertal and pubertal subgroups. Robustness of the meta-analysis for each outcome was assessed by sensitivity analysis, carried out with the Comprehensive Meta-Analysis software, that was run by eliminating studies one-by-one, and differences in estimations above 0.5 mm (for linear outcomes) or 0.5° (for angular outcomes) were considered as clinically relevant. Moreover, the overall quality of evidence for each of the primary outcomes, according to the pre-pubertal and pubertal subgroups, was evaluated following the Grades of Recommendation, Assessment, Development, and Evaluation (GRADE) guidelines using the GRADE profiler software version 3.6.1 (www.gradeworkinggroup.org) [30]. The GRADE assesses the quality of evidence as high, moderate, low and very low based on eight different domains as follows: i) risk of bias, ii) inconsistency, iii) indirectness, iv) imprecision, v) publication bias, vi) large effect, vii) plausible confounding that would change effect, and viii) dose response gradient [31]. Although the GRADE has been developed for RCTs, also CCTs were entered in the profiler software as randomised studies, provided that they were downgraded by 1 point in the ‘risk of bias’ domain. All the other GRADE domains were filled according to the published recommendations [30] with the exception of the ‘large effect’ domain score that was determined on data regarding differential growth increment in untreated Class II and Class I subjects [32]. In particular, the mean annualised changes for the cephalometric measurements in the pre-pubertal and pubertal subjects were derived from this reported growth study [32]. Subsequently, 1 mm was added to account for the cephalometric method error, as this value may be considered representative of linear cephalometric error measurements. Therefore, by a slight excess approximation the large effects were set as 1.5 mm/year for all the primary outcomes for pre-pubertal patients, and as 2.5 mm/year for total and composite mandibular length (Pancherz analysis), and as 2.0 mm/year and 1.5 mm/year for the mandibular ramus height and mandibular base (Pancherz analysis), respectively, in pubertal patients. A very large effect was set by adding 1 mm to each threshold. Moreover, due to the lack of reporting for the composite mandibular length and mandibular base (Pancherz analysis), the total mandibular length and Pogonion to Nasion perpendicular [32], respectively, were used instead to elaborate dimensions of the effect.

## Results

### Study search

**Fig 1:**
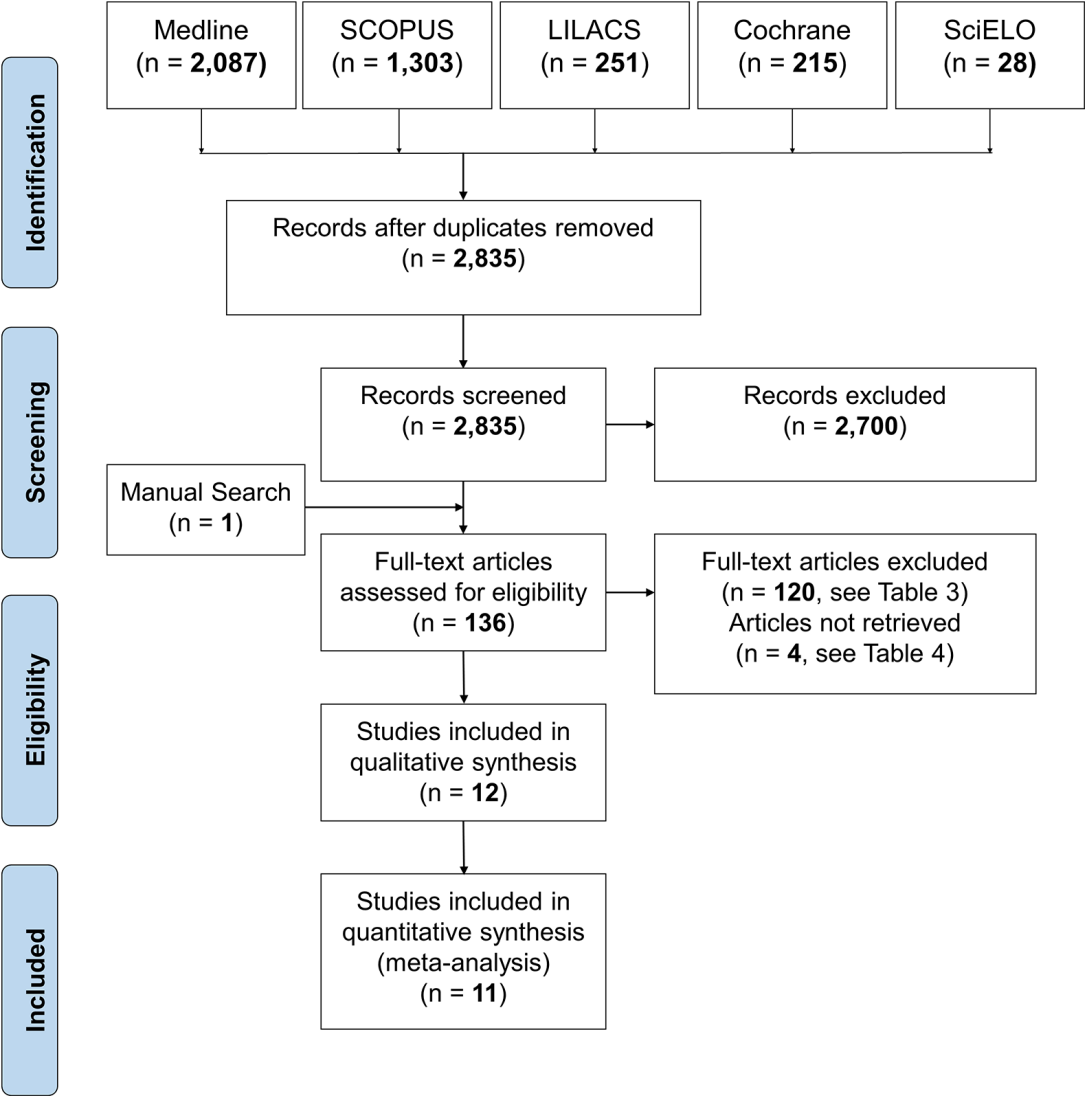
Flow diagram of the search strategy.

The results of the electronic and manual searches are summarised in Fig 1. According to the electronic search, a total of 2,458 articles were retrieved. Among these, 12 studies [33–44] were judged to be relevant to the present review. However, 2 articles were clearly derived from the same study sample reporting either the results about soft tissues and SNA, SNB and ANB angles [41] or other dentoskeletal effects [44] and may be considered as a single study. Full details of the excluded studies at the full text analysis are reported in the Table 3. Four studies could not be retrieved upon internet search, through the local library facility, and after having contacted the Authors (Table 4). Finally, 1 study [40] included in the qualitative synthesis, was not included in the meta-analysis according to the risk of bias and sensitivity analyses (see below).

**Table 3:** Studies excluded after full text consideration with corresponding main reason of exclusion.

Authors	Year	Reference	Main Reason for exclusion
**1.** Jakobsson	1967	Am J Orthod 53:446–57	No skeletal maturation evaluation
**2.** Freunthaller	1967	Angle Orthod 37:18–22	No skeletal maturation evaluation
**3.** Fraenkel	1969	Am J Orthod 55:265–75	No skeletal maturation evaluation
**4.** Luedtke	1973	Am J Orthod 63:18–29	No skeletal maturation evaluation
**5.** Lewis	1976	Am J Orthod 70: 529–549	Case series
**6.** Bernstein *et al*.	1976	Am J Orthod 70:683–9	No skeletal maturation evaluation
**7.** Ahlgern and Laurin	1976	Br J Orthod 3:181–7	No control group
**8.** Bernstein *et al*.	1977	Am J Orthod 72: 549–559	Headgear treatment
**9.** Brunner	1979	Rev Orthop Dento Facial 13:269–73	Expert opinion
**10.** Wieslander and Lagerström	1979	Am J Orthod 79:20–6	No skeletal maturation evaluation
**11.** Bonnefont and Charron	1979	Rev Orthop Dento Facial 13:39–48	No skeletal maturation evaluation
**12.** Luder	1981	Eur J Orthod 3:205–22	No skeletal maturation evaluation
**13.** Baumrind and Korn	1981	Am J Orthod 80:31–47	No skeletal maturation evaluation
**14.** Cohen	1981	Br J Orthod 8:159–63	No skeletal maturation evaluation
**15.** Klaassen	1981	J Oral Surg 39:849–54	Case report
**16.** Luder	1982	Am J Orthod 81:390–6	No skeletal maturation evaluation
**17.** Calvert	1982	Br J Orthod 9:149–53	No skeletal maturation evaluation
**18.** Baumrind *et al*.	1983	Am J Orthod 84:443–65	No skeletal maturation evaluation
**19.** Choroschilkina and Malygin	1984	1984 Fortsch Kieferorthop 45 448–4	No skeletal maturation evaluation
**20.** Brieden *et al*.	1984	Angle Orthod 54:226–32	Not reporting primary outcomes
**21.** Madone and Ingervall	1984	Eur J Orthod 6:92–106	Not including a control group
**22.** Vargervik and Harvold	1985	Am J Orthod 88:242–51	No skeletal maturation evaluation
**23.** McNamara *et al*.	1985	Am J Orthod 88:91–110	No skeletal maturation evaluation
**24.** Haynes	1986	Angle Orthod 56:309–14	No skeletal maturation evaluation
**25.** Haynes	1986	Am J Orthod Dentofacial Orthop 90:308–20	No skeletal maturation evaluation
**26.** Stefani and Munster	1987	Fortschr Kieferorthop 48:154–60	Case series
**27.** DeVincenzo *et al*.	1987	Am J Orthod Dentofacial Orthop 91:213–24	No skeletal maturation evaluation
**28.** Fjlgen *et al*.	1987	Fortschr Kieferorthop 48:41–51	No skeletal maturation evaluation
**29.** Enlow *et al*.	1988	Eur J Orthod 10:192–202	No skeletal maturation evaluation
**30.** Falck and Frankel	1989	Am J Orthod Dentofacial Orthop 96:333–41	No skeletal maturation evaluation
**31.** DeVincenzo and Winn	1989	Am J Orthod Dentofacial Orthop 96:181–90	No skeletal maturation evaluation
**32.** Kerr *et al*.	1989	Eur J Orthod 11:235–42	No skeletal maturation evaluation
**33.** Dahan *et al*.	1989	Am J Orthod Dentofacial Orthop 95:127–37	No skeletal maturation evaluation
**34.** McNamara *et al*.	1990	Am J Orthod Dentofacial Orthop 98:134–44	No skeletal maturation evaluation
**35.** Stüber	1990	Fortschr Kieferorthop 51:361–5	No skeletal maturation evaluation
**36.** Jakobsson and Paulin	1990	Eur J Orthod 12:174–84	No skeletal maturation evaluation
**37.** Derringer	1990	Br J Orthod 17:33–46	No skeletal maturation evaluation
**38.** Drage and Kunt	1990	Br J Orthod 17:205–13	Limited to successful cases
**39.** Falck	1991	1991 Fortschr Kieferorthop 52:263–7	No skeletal maturation evaluation
**40.** Falck and Zimmermann	1991	1991 Fortschr Kieferorthop 52:98–101	No skeletal maturation evaluation
**41.** Ball and Hunt	1991	Eur J Orthod 13:53–8	No skeletal maturation evaluation
**42.** Ball and Hunt	1991	Eur J Orthod 13:47–52	No skeletal maturation evaluation
**43.** Nelson *et al*.	1993	Am J Orthod Dentofacial Orthop 104:153–61	No skeletal maturation evaluation
**44.** Vaden *et al*.	1995	Am J Orthod Dentofacial Orthop 107:651–61	Mixing different treatment modalities
**45.** Sander and Wichelhaus	1995	1995 Fortschr Kieferorthop 56:127–39	No skeletal maturation evaluation
**46.** Lange *et al*.	1995	Angle Orthod 65:423–30	No skeletal maturation evaluation
**47.** Webster *et al*.	1996	Am J Orthod Dentofacial Orthop 110:46–53	No skeletal maturation evaluation
**48.** Courtney *et al*.	1996	Am J Orthod Dentofacial Orthop 109:616–24	No skeletal maturation evaluation
**49.** Battagel	1996	Eur J Orthod 18:41–54	No skeletal maturation evaluation
**50.** Perillo *et al*.	1996	Am J Orthod Dentofacial Orthop 109:132–9	No skeletal maturation evaluation
**51.** Kumar *et al*.	1996	J Clin Pediatr Dent 20:101–8	No skeletal maturation evaluation
**52.** Cura and Sarac	1997	Eur J Orthod 19:691–702	Mixing different treatment modalities
**53.** de Oliveira and de Oliveira	1997	Journal Brasileiro de Odontologia Clinica 1:51–63	No skeletal maturation evaluation
**54.** Illing *et al*.	1998	Eur J Orthod 20:501–16	No skeletal maturation evaluation
**55.** Mills and McCulloch	1998	Am J Orthod Dentofacial Orthop 114:15–24	No skeletal maturation evaluation
**56.** Lund *et al*.	1998	Am J Orthod Dentofacial Orthop113: 104–110	No skeletal maturation evaluation
**57.** Keeling *et al*.	1998	Am J Orthod Dentofacial Orthop 113:40–50	No skeletal maturation evaluation
**58.** Özbek *et al*.	1998	Angle Orthod 68:327–336	Unclear skeletal maturation assessment/distribution
**59.** Toth and McNamara	1999	Am J Orthod Dentofacial Orthop 116:597–609	No skeletal maturation evaluation
**60.** Tümer and Gültan	1999	Am J Orthod Dentofacial Orthop 116:460–8	Unclear skeletal maturation assessment/distribution
**61.** Rushfordt *et al*.	1999	Br J Orthod 26:127–34	No skeletal maturation evaluation
**62.** Lai *et al*.	1999	Hua Xi Kou Qiang Yi Xue Za Zhi 17:271–4	Not including a control group
**63.** Ehmer *et al*.	1999	J Orofac Orthop 60:392–408	Redundant publication
**64.** Mills and McCulloch	2000	Am J Orthod Dentofacial Orthop 118:24–33	Limited to successful cases
**65.** Trenauth	2000	Am J Orthod Dentofacial Orthop 117:54–59	No skeletal maturation evaluation
**66.** Trenauth *et al*.	2001	J Orofac Orthop 62:466–75	No skeletal maturation evaluation
**67.** Chadwick et al.	2001	Eur J Orthod 23:495–505	No skeletal maturation evaluation
**68.** Vardimon et al.	2001	Am J Orthod Dentofacial Orthop 120:416–26	No skeletal maturation evaluation
**69.** Eckardt et al.	2001	J Orofac Orthop 62:337–49	No skeletal maturation evaluation
**70.** Üçüncü et al.	2001	J Orofac Orthop 62:224–37	No skeletal maturation evaluation
**71.** Lux et al.	2001	Angle Orthod 71:120–6	No skeletal maturation evaluation
**72.** Ruf et al.	2001	Angle Orthod 71:4–11	Inappropriate control group
**73.** de Almeida	2001	R Dental Press Ortodon Ortop Facial 6:11–27	No skeletal maturation evaluation
**74.** Trenauth	2002	Eur J Orthod 24:485–91	Inappropriate control group
**75.** de Almeida et al.	2002	Angle Orthod 72:418–25	No skeletal maturation evaluation
**76.** de Almeida et al.	2002	Am J Orthod Dentofacial Orthop 121:458–66	No skeletal maturation evaluation
**77.** Wheeler et al.	2002	Am J Orthod Dentofacial Orthop 121:9–17	Unclear skeletal maturation assessment/distribution
**78.** Oliveira	2002	R Dental Press Ortodon Ortop Facial 7:55–63	No skeletal maturation evaluation
**79.** Janson et al.	2003	Eur J Orthod 25:301–9	No skeletal maturation evaluation
**80.** Basciftci et al.	2003	Eur J Orthod 25:87–93	No skeletal maturation evaluation
**81.** Cevidanes et al.	2003	Am J Orthod Dentofacial Orthop 123:379–87	Not reporting primary outcomes
**82.** Cozza et al.	2004	Angle Orthod 74:741–48	No skeletal maturation evaluation
**83.** Araujo et al.	2004	Am J Orthod Dentofacial Orthop 126:666–71	No skeletal maturation evaluation
**84.** Araujo et al.	2004	Eur J Orthod 26:515–22	No skeletal maturation evaluation
**85.** Cozza et al.	2004	Eur J Orthod 26:293–302	No skeletal maturation evaluation
**86.** Tulloch et al.	2004	Am J Orthod Dentofacial Orthop 125:657–67	Second phase of an already included study
**87.** Almeida et al.	2004	Eur J Orthod 26:65–72	No skeletal maturation evaluation
**88.** Jena et al.	2005	J Clin Pediart Dent 29:225–30	No skeletal maturation evaluation
**89.** Šidlauskas	2005	Stomatologija 7:7–10	No skeletal maturation evaluation
**90.** Cevidanes et al.	2005	Am J Orthod Dentofacial Orthop 128:16–26	Not reporting primary outcomes
**91.** Cevidanes et al.	2005	Am J Orthod Dentofacial Orthop 128:27–34	Not reporting primary outcomes
**92.** Wedler et al.	2006	J Orofac Orthop 67:105–15	No skeletal maturation evaluation
**93.** Türkkahraman and Özgür	2006	Eur J Orthod 28:27–34	No skeletal maturation evaluation
**94.** Jena et al.	2006	Am J Orthod Dentofacial Orthop 130:594–602	Not reporting primary outcomes
**95.** Dolce et al.	2007	Am J Orthod Dentofacial Orthop 132:481–9	No skeletal maturation evaluation
**96.** Parsekian Martins et al.	2008	Am J Orthod Dentofacial Orthop 134:732–41	No skeletal maturation evaluation
**97.** Woods	2008	Am J Orthod Dentofacial Orthop 133:388–94	No skeletal maturation evaluation
**98.** Varlik et al.	2008	Eur J Orthod 30:128–34	Not reporting primary outcomes
**99.** O'Brien	2009	Am J Orthod Dentofacial Orthop 135:573–9	No skeletal maturation evaluation
**100.** Jena and Duggal	2010	Angle Orthod 80:485–91	No skeletal maturation evaluation
**101.** Baccetti and McNamara	2010	Prog Orthod 11:118–26	Mixing different treatment modalities
**102.** Siara-Olds et al.	2010	Angle Orthod 80:18–29	Unclear skeletal maturation assessment/distribution
**103.** Malta et al.	2010	Angle Orthod 80:10–7	Unclear skeletal maturation assessment/distribution
**104.** Li et al.	2010	Hua Xi Kou Qiang Yi Xue Za Zhi 28:637–40	Not reporting primary outcomes
**105.** Nedeljkovic	2011	Principles in Contemporary Orthodontics: 79–112	No skeletal maturation evaluation
**106.** Perillo et al.	2011	Eur J Pediatr Dent 12:261–6	No short term effects evaluated
**107.** Barros Nunes et al.	2011	Orthod Sci Pract 3:517–23	No skeletal maturation evaluation
**108.** Brunharo et al.	2011	Dental Press J Orthod 16:40.e1-8	Samples apparently included in another included study
**109.** Mahamad et al.	2012	Int J Orthod Milwaukee 23:49–58	No skeletal maturation evaluation
**110.** Alió-Sanz et al.	2012	Med Oral Patol Oral Cir Bucal 17:e884-92	Skeletal maturation assessment not valid
**111.** Alió-Sanz et al.	2012	Med Oral Patol Oral Cir Bucal 17:e689-96	Not reporting primary outcomes
**112.** Silvestrini-Biavati et al.	2012	Eur J Pediatr Dent 13:301–6	Unappropriated control group
**113.** Singh et al.	2012	J of Oral Biol Craniofac Res 2:61–66	Case series
**114.** Pieri et al.	2012	OrtodontiaSPO 45:525–36	Inconsistent durations of treatment and observational terms
**115.** Jena et al.	2013	Angle Orthod 83:728–734	No skeletal maturation evaluation
**116.** Antunes et al.	2013	Angle Orthod 83:455–9	Not reporting primary outcomes
**117.** Uzuner et al.	2014	J Orofac Orthop 75:275–86	No skeletal maturation evaluation
**118.** Saikoski et al.	2014	Dental Press J Orthod 19:36–45	No skeletal maturation evaluation
**119.** Bigliazzi et al.	2014	Angle Orthod 3 Dec Epub	Not reporting primary outcomes
**120.** Giuntini et al.	2015	Angle Orthod 18 Mar Epub	Mixing different skeletal maturation phases into same group

**Table 4:** Studies that could not be retrieved for full text analysis.

**1.** Falck F. [Sagittal and vertical changes in mandibular retrognathism. A teleradiological longitudinal study of patients with functional regulators compared to a control group]. Stomatol DDR. 1983;33:182–95. Article in German.
**2.** Demisch A. [Long-term observation of the occlusal stability after distal bite therapy with the Bern activator]. SSO Schweiz Monatsschr Zahnheilkd. 1980;90:867–80. Article in German.
**3.** Parkhonse RC. A cephalornetric appraisal of cases of Angle's Class II, division 1 malocclusion treated by the Andresen appliance. Trans Br Soc Study Orthod1969;55:61–70.
**4.** Lucchese A, Carinci F, Brunelli G. Skeletal effects induced by twin block in therapy of class II malocclusion. Eur J Inflamm; 2012;10:83–87.

The only study (Lucchese et al. 2012) for which the abstract could be retrieved examined the skeletal effects of the Twin-Block appliance treatment in pubertal subjects, with findings similar to those of the other investigations included herein.

### Study design and treatment interventions

Details on study designs and interventions of the included studies are summarised in Table 5. A total of three RCTs were retrieved, including both pre-pubertal [33], [36] and pubertal [42] subjects. Four studies [33], [36], [38], [43] included only pre-pubertal subjects, 4 more studies [37], [39], [41], [42], [44] included only pubertal subjects, and 3 studies [34], [35], [40] included both pre-pubertal and pubertal subjects. The enrolment of the treated group was prospective in 6 studies [33], [36–38], [41], [42], [44], and retrospective in the rest of the studies.

**Table 5:** Protocols of the studies included in the present systematic review.

Study	Study design	Sample size; age in yrs as mean± SD (or range)	Appliance	Class II description	Skeletal maturation method/stages	Prognostic or other features	Cephalometric magnification factor	Treatment or observation duration/ Appliance wear	Mandibular advancement /treatment stopped
**Pre-pubertal subjects**									
Tulloch et al. [33]	RCT	23 F; 30 M; 9.4 ±1.0	Bionator	Overjet≥7 mm	HWM/ at least 1 year pre-peak	All permanent incisors and first molars erupted	NA	1.3 yrs/ NA	4–6 mm mandibular advancement with minimal vertical opening/ At 15 months (and continued if clinical objectives were not achieved)
		26 F;35 M; 9.4 ±1.2	Control					1.3 yrs/ —	
Baccetti et al. [34]	CCT, R	11 F; 10 M; 9.0 ±0.9	Twin-Block	Full Class II molar relationship	CVM/ CS 1 to 2	NA	0%	1.2 ±0.3 yrs/ Full time	Mandibular advancement to an incisor end-to-end (except for patients with overjet>7 mm in whom 4–6 mm initial advancement was performed) with 5–7 mm vertical posterior opening/ NA
		7 F; 9 M; 9.1 ±0.8	Control					1.3 ±0.6 yrs/ —	
Faltin et al. [35]	CCT, R	7 F; 6 M; 9.7 ±1.3	Bionator	Full Class II molar relationship	CVM/ CS 1 to 2 at end of treatment	NA	0%	1.8 ±0.6 yrs/ NA	NA
		5 F; 6M; 9.4±1.3	Control		CVM/ CS 1 to 2			2.1 ±0.6 yrs/ —	
O’Brien et al. [36]	RCT	41 F; 48 M; 9.7±0.98	Twin-Block	Overjet >7 mm	CVM/ pre-peak	NA	0%	1.25 yrs/ Full time	7–8 mm mandibular advancement/ When overjet was fully reduced
		39 F; 46 M; 9.8±0.94	Control					1.25 yrs/ —	
Almeida-Pedrin et al. [38]	CCT, P	15 F; 15M; 10.35 (8.2–11.0)	Bionator	Class II/1; bilateral molar Class II relationship greater than one-half cusp; ANB ≥4.5°	CVM/ CS 1 to 2	NA	9%	1.52 yrs/ NA	NA
		15 F; 15M; 10.0 (8.0–10.9)	Control					1.49 yrs/ —	
Singh et al. [40]	CCT, R	5 (NA); NA	Twin-Block	Class II/1; Full Class II molar relationship on one side and end-on or greater on the other side; retrognathic mandible; ANB >4°	CVM/ CS 1 to 2	Normal maxillary position; normal to horizontal growth pattern with little or no vertical problems	0%	1 yr, **a**/ Full time	Mandibular advancement of 10 mm with the interincisal clearance of 2 mm (except for patients with overjet>10 mm in whom 7–8 mm initial advancement was performed, then a second activation), **a**/ NA
		5 (NA); NA	Control					2 yrs/ —	
Perillo et al. [43]	CCT, R	9 F; 8 M, a; 8.9±1.1	FR-2	Class II/1; Full or half-cusp Class II molar relationship; overjet >4 mm; ANB >4°; SNB <78°	MPM/ MPS 1 to 2	No maxillary protrusion	8%	1.6 ± 0.8 yrs/ 18 h per day	Mandibular advancement less than 3 mm/ At full Class I molar relationship
		10 F; 7 M, a; 8.9±1.8	Control					1.6 ± 0.8 yrs/ —	
**Pubertal subjects**									
Baccetti et al. [34]	CCT, R	6 F; 9 M; 12.9±1.2	Twin-Block	Full Class II molar relationship	CVM/ CS 3 to 5	NA	0%	1.4 ±0.4 yrs/ Full time	Mandibular advancement to an incisor end-to-end (except for patients with overjet>7 mm in whom 4–6 mm initial advancement was performed, then a second activation). Also, 5–7 mm vertical posterior opening/ NA
		7 F; 7 M; 13.6±1.2	Control					1.3 ±0.4 yrs/ —	
Faltin et al. [35]	CCT, R	6 F; 4 M; 10.8±1.7	Bionator	Full Class II molar relationship	CVM/ CS 3 or 4 at end of treatment	NA	0%	2.3 ±1.5 yrs/ NA	NA
		5 F; 5 M; 11.2±1.5	Control		CVM/ CS 3 or 4			1.8 ±0.7 yrs/ —	
Quintão et al. [37]	CCT,P	7 F; 12 M; 9.5±0.8	Twin Block	Class II/1 with distal canine and molar relationship; ANB > 4 degrees; overjet ≥ 6 mm	Epiphyseal stages FP, FM, G1 and Psi	NA	NA	1.0±0.08 yrs/ full time	4 mm mandibular advancement with re-activation after 6 months if needed/ After 12 months
		7 F; 12 M; 9.9±1.1	Control					1.0±0.08 yrs/ —	
Cui et al. [39]	CCT, R	9 F; 18 M; 11.7 (NA)	Twin Block	Class II/1; distal molar relationship; overjet ≥4 mm; ANB ≥5°	HWM/ NA	Deep bite	NA	1.2 yrs/ NA	NA
		9 F; 12 M; 11.3 (NA)	Control					1.2 yrs/ —	
Singh et al. [40]	CCT, R	29 (NA); NA	Twin-Block	Class II/1; full Class II molar relationship on one side and end-on or greater on the other side; retrognathic mandible; ANB >4°	CVM/ CS 3 to 4	Normal maxillary position; normal to horizontal growth pattern with little or no vertical problems	0%	1 yr, a/ Full time	Mandibular advancement of 10 mm with the interincisal clearance of 2 mm (except for patients with overjet>10 mm in whom 7–8 mm initial advancement was performed, then a second activation), **a** /NA
		29 (NA); NA	Control					2 yrs/ —	
Martina et al. [42]	RCT	8 F; 15 M; 10.9±1.3	Sander Bite Jumping	Full Class II molar relationship; overjet ≥6 mm	CVM/ CS 3	SN-MP angle smaller than normal value ±SD	0%	1.5 yrs/ 14h per day	Initial 4 mm mandibular advancement followed by individual 1.5 mm advancements/ At full Class I molar relationship; maximum treatment duration set at 1.5 yrs
		12 F; 11 M; 10.5±1.2	Control					1 yr/—	
Baysal and Uysal [41], [44]	CCT, P	10 F, 10 M; 13.0 ±1.3	Twin Block	SNB <78°; ANB >4°; overjet ≥5 mm; bilateral molar Class II relationship (at least 3.5 mm)	HWM/ Fourth (S and H2) or fifth (MP3cap) epiphyseal stages	SN-GoGn angle of 32°±6°	14%, a	1.3 ±0.6/ Full time	Mandibular advancement by 70% of the maximum protrusive path, then a second activation/ At normal or overcorrected overjet
		9 F, 11 M; 12.2 ±1.5	Control					1.3 ±0.3 yrs/—	

**RCT**, randomized clinical trial; **CCT**, controlled clinical trial; **P**, prospective; **R**, retrospective; **NA**, not available; **F**, females; **M**, males; **CVM**, cervical vertebral maturation; **CS**, CVM stage; **HWM**, hand-and-wrist maturation; **MP3cap**, medial phalanx capping stage of the third finger; **FMA**, Frankfurt/mandibular plane angle; **MP**, mandibular plane.

**a**, information provided by the Authors; —, not applicable.

The sample size per group ranged from a minimum of 5 [40] to a maximum of 89 [36] for the pre-pubertal groups, and from 10 [35] to 29 [40] for the pubertal groups. The mean subjects age ranged from 8.9 [43] to 10.3 [38] years for the pre-pubertal groups, and from 9.5 [37] to 14.0 [34], for the pubertal groups. All the studies included both male and female subjects. The removable functional appliances used were Twin-Block [34], [36], [37], [39–41], [44], Bionator [33], [35], [38], Function regulator type 2 (FR-2) [43] and Sander bite jumping [42]. Two RCTs [33], [36] assessed Class II malocclusion only on the basis of an overjet ≥7 mm, 3 studies [34], [35], [42] included subjects that had to have a full Class II molar relationship, the rest of the studies [37–41], [43], [44] generally assessed Class II malocclusion by a combination of ANB angle >4° (at least) and Class II molar relationship. To assess growth phase, 6 studies used the CVM method [34–36], [38], [40], [42], while the rest used various HWM method [33], [37], [39], [41], [43], [44]. Four studies, 2 on pre-pubertal [40], [43] and 2 on pubertal subjects [40], [42], reported a normal sagittal position of the maxilla in the included patients. One study [40] on both pre-pubertal and pubertal patients, and more 3 studies [40], [42], [44] on pubertal patients reported an absence of vertical facial growth. The rest of the studies did not report any further prognostic or diagnostic feature, with the exception of some dental maturation stage [33] or presence of deep bite [39]. Cephalometric magnifications were set at 0% [34–36], [40], [42], 8–9% [38], [43], or 14% [41], [44]. In the rest of the studies, information was not provided. The mean treatment duration for the pre-pubertal subjects ranged from 1 year [40] to 2.1 years [35] with the appliance being worn at least 18 hours per day [43] to full time wear [34], [36], [40]. However, 3 studies [33], [35], [38] on pre-pubertal subjects did not report any information about appliance wear in terms of hours per day.

The mean treatment duration in the pubertal subjects ranged from 1 year [37] to 1.8 years [35], with the appliance being worn at least 14 hours per day [42] to full time wear [34], [37], [40]. Two studies [35], [39] on pubertal subjects did not report the mean appliance wear time. In 1 study [40] including both pre-pubertal and pubertal subjects, treatment duration lasted for 1 year although post-treatment measurements were performed after an additional year of retention. Generally, a single mandibular advancement to an incisor end-to-end relationship was performed for overjet up to 7–10 mm; otherwise, a 2-step procedure was followed [33], [34], [36], [37], [40], [42]. Mandibular advancement by 70% of the maximum protrusive path was used in 1 study [41], [44]. Furthermore, a stepwise advancement of less than 3 mm was performed in one study [43]. Other studies did not report the amount of mandibular advancement during treatment. Treatment was stopped when a Class I molar relationship [42], [43], or a normal overjet was achieved in a mandibular retruded position [36], [41], [44]. In 1 RCT [33] treatment was performed for at least 15 months and continued if clinical objectives were not achieved. The rest of the studies did not report when treatment was stopped.

### Main results

Main results in the included studies are summarised in Table 6. On pre-pubertal patients, 1 RCT and 1 CCT reported improvement in 75% [33] and 65% [43] of the cases. Another study on pubertal patients reported a 100% success rate [41], [44], while in the rest of the studies the treatment success rate was not reported. Significant skeletal effects were reported mainly in the studies including pubertal subjects, even though 3 studies including pre-pubertal subjects reported a significant increase of mandibular length [33], [34], or growth modification at the maxillary level [36] that would be of poor clinical meaning. On the contrary, 3 studies including pre-pubertal subjects reported no skeletal effects [35], [40], [43]. All the studies including pubertal subjects reported a significant increase of mandibular length [34], [35], [37], [39–42], [44], an opening of the gonial angle [34], [35], an increase of lower anterior facial height [40], [41], [44], and maxillary growth restrain [41], [44]. Regardless of the growth phase, dentoalveolar effects were generally seen. Six studies [33–36], [40], [43] on pre-pubertal patients concluded that treatment had no or minimal skeletal effects. On the contrary, 6 studies including pubertal patients reported that optimal timing for functional treatment would be during or slightly after the pubertal growth spurt [34], [35], [37], [40], [42]. Finally, 2 studies on pre-pubertal [38] and pubertal patients [41], [44] did not comment on treatment timing. At the mandibular level, these effects were reported as mesial movement of the mandibular dentition [34–36], [38], [40], extrusion of lower first molars [38], and proclination of lower incisors [34], [38], [39], [42]. At the maxillary level, dentoalveolar treatment effects as reclination of upper incisors were reported both in pre-pubertal [43] and pubertal patients [37–40], [42]. One study [34] reported distal movement of the maxillary dentition, and a further study [40] reported absence of any dentoalveolar treatment effects for the pre-pubertal subjects. The rest of the studies did not report information about dentoalveolar effects at the maxillary level. Modifications of the soft tissue profile were described in only 4 studies [37], [39–41], [44] on pubertal patient as improvement of the profile, mainly due to soft tissue Pogonion advancement [37], [40], [41], [44], upper lip retraction [37], [39], or lower lip protraction [40] were also reported. On the contrary, 1 study [40] including pre-pubertal subjects reported no significant soft tissue changes.

**Table 6:** Treatment effects in the studies included in the present systematic review.

Study	Success rate, a	Main treatment effects			Conclusions on growth phase and treatment efficiency
		**Skeletal**	Dentoalveolar	Soft tissues	
**Pre-pubertal subjects**					
Tulloch et al. [33]	75% improved cases	Significant increase in mandibular length and protrusion	Overjet and overbite reduction	NA	Early functional treatment reduces the severity of Class II skeletal pattern. Children with Class II malocclusion experience considerable variation in growth
Baccetti et al. [34]	NA	Significant mandibular length increase and significant opening of gonial angle	Overjet reduction, mesial movement of maxillary and distal movement of mandibular molars, mandibular incisor proclination	NA	Optimal timing for functional treatment appears to be during or slightly after the onset of the pubertal peak
Faltin et al. [35]	NA	No significant skeletal effects	Significant overjet reduction and correction of molar relationship	NA	No significant skeletal effects of functional treatment performed during the pre-pubertal growth phase
O’Brien et al. [36]	NA	Significant skeletal growth modification at maxillary and mandibular level, however not clinically relevant	Overjet reduction and correction of molar relationship	NA	Early functional treatment reduces overjet in Class II malocclusion patients mainly due to dentoalveolar changes, with clinically insignificant skeletal effects
Almeida-Pedrin et al. [38]	NA	Significant increase in mandibular protrusion, but not in mandibular length	Significant maxillary incisor retrusion and reclination, and mandibular incisor protrusion and proclination, extrusion and mesial movement of mandibular molars	NA	None
Singh et al. [40]	NA	No significant skeletal effects	No significant dental effects	No significant soft tissue effects	Optimal timing for functional treatment would be during or slightly after the pubertal growth spurt
Perillo et al. [43]	65%, b	No significant skeletal effects, except for ANB angle reduction	Significant overjet reduction, maxillary incisor reclination	NA	Treatment at the pre-pubertal growth phase has no mandibular effects
**Pubertal subjects**					
Baccetti et al. [34]	NA	Significant mandibular length increase and significant opening of gonial angle	Overjet reduction, mesial movement of maxillary and distal movement of mandibular molars, mandibular incisor proclination	NA	Optimal timing for functional treatment appears to be during or slightly after the onset of the pubertal peak
Faltin et al. [35]	NA	Significant increase in mandibular length and ramus height with opening of the gonial angle	Significant overjet reduction and correction of molar relationship with mesial movement of the mandibular dentition	NA	Optimal timing to start functional treatment is immediately before the pubertal growth spurt.
Quintão et al. [37]	NA	Significant increase of mandibular length and ANB reduction	Upper incisor reclination and overjet reduction	Upper lip retraction and forward advancement of soft tissue Pogonion	A relevant degree of skeletal correction could be obtained at pubertal stage of development. However, an extended growth period would be needed for a complete Class II correction.
Cui et al. [39]	NA	Significant increase in mandibular length	Significant overjet reduction, maxillary incisor reclination, lower incisor proclination	Upper lip retraction and reduction of mentolabial sulcus angle	Functional treatment for Class II malocclusion at the pubertal growth spurt improves relationship of basal bones
Singh et al. [40]	NA	Significant increase in mandibular protrusion and length; significant increase in lower anterior facial height	Significant overjet and overbite reduction; maxillary incisor reclination, extrusion and mesial movement of mandibular molars	Significant advancement of lower lip and soft tissue Pogonion	Optimal timing for functional treatment of Class II malocclusion is during or slightly after the pubertal growth spurt.
Martina et al. [42]	NA	Significant increase in mandibular length	Significant overjet reduction, maxillary incisor reclination and mandibular incisor proclination		Treatment response was relevant and not influenced by the cervical stage (3 or 4) among pubertal subjects
Baysal and Uysal [41], [44]	100%	Increase in composite mandibular length, maxillary growth restrain, significant increase in lower anterior and posterior face heights	Overjet reduction	Significant advancement of soft tissue Pogonion and lower lip	None

**NA**, not available

**a**, as defined by the authors

**b**, information provided by the Authors.

### Risk of bias in individual studies

Detailed information on the risk of bias in individual studies is shown in Tables 7 and 8 for the RCTs and CCTs, respectively. Briefly, 2 RCTs [33], [36] had an unclear bias with regard to the diagnosis of Class II malocclusion based on the overjet alone, while the last RCT [42] did not show significant risk of bias. Regarding the CCTs, the overall scores ranged from 12 [40] to 24 [41], [43], [44]. Only 1 study had an overall score below the threshold and was thus judged as affected by significant risk of bias [40], two studies [37], [39] reached 15 points, 1 study [38] reached 16 points, 1 study [35] reached 19 points, and the last 1 study [34] reached 20 points.

**Table 7:** Risk of bias for the randomised clinical trials according to the Cochrane tool.

Study	Random sequence generation (selection bias)	Allocation concealment (selection bias)	Blinding of personnel (performance bias), a	Blinding of outcome assessment (detection bias)	Incomplete outcome data (attrition bias)	Selective reporting (reporting bias)	Other bias	****Overall risk of bias****
Tulloch et al. [33]	Low	Unclear	Low	Unclear	Low	Low	Unclear, **b**	Unclear
O’Brien et al. [36]	Low	Low	Low	Low	Low	Low	Unclear, **b**	Unclear
Martina et al. [42]	Low	Low	Low	Low	Low	Low	Low	Low

**a**, Even if not feasible, the risk of bias for non-blinded personnel performing the treatment was not judged as a significant risk of bias

**b**, Class II malocclusion determined only by overjet.

**Table 8:** Risk of bias for the controlled clinical trials according to the modified Downs and Black tool.

****Item****	Baccetti et al. [34]	Faltin et al. [35]	Quintão et al. [37]	Almeida-Pedrin et al. [38]	Cui et al. [39]	Singh et al. [40]	Baysal and Uysal [41], [44]	Perillo et al. [43]
**Reporting**								
**1.** Is the objective of the study clearly described?	Yes	Yes	Yes	Yes	Yes	Yes	Yes	Yes
**2.** Are the main outcomes to be measured clearly described in the Introduction or Methods section (including cephalometric magnification)?	Yes	Yes	No	Yes	Yes	Yes	Yes	Yes
**3.** Were inclusion and exclusion criteria clearly stated?	Yes	Yes	No	No	No	Yes	Yes	Yes
**4.** Are the characteristics of the patients included clearly described?	Yes	No	Yes	Yes	No	No	Yes	Yes
**5.** Is the Class II malocclusion fully described?	No	No	Yes	Yes	Yes	Yes	Yes	Yes
**6.** Are the interventions of interest clearly described?	Yes	No	Yes	No	No	No	Yes	Yes
**7.** Are the distributors of principal confounders in each group of subjects to be compared clearly described?	Yes	Yes	Partially	Partially	Partially	No	Yes	Yes
**8.** Are the main findings of the study clearly described?	Yes	Yes	Yes	Yes	Yes	Yes	Yes	Yes
**9.** Does the study provide estimates of the random variability in the data for the main outcomes?	Yes	Yes	Yes	Yes	Yes	Yes	Yes	Yes
**10.** Have all important adverse events that may be a consequence of functional appliances been reported?	Yes	Yes	Yes	Yes	Yes	Yes	Yes	Yes
**11.** Have the characteristics of patients lost to follow-up been described?	Yes	Yes	Yes	Yes	Yes	Yes	Yes	Yes
**12.** Have actual probability values been reported for the main outcomes except where the probability value is less than 0.001?	No	No	No	No	Yes	No	Yes	No
**External validity**								
**13.** Were the patients asked to participate in the study representative of the entire population from which they were recruited?	Yes	Yes	Unclear	Unclear	Unclear	Unclear	Yes	Yes
**14.** Were those subjects who were prepared to participate representative of the entire population from which they were recruited?	Unclear	Unclear	Unclear	Unclear	Unclear	Unclear	Yes	Yes
**15.** Were the staff, places, and facilities where the patients were treated, representative of the treatment the majority of patients receive?	Yes	Yes	Yes	Yes	Yes	Yes	Yes	Yes
**Internal validity—bias**								
**16.** Was an attempt made to blind those measuring the main outcome of the intervention?	No	No	Unclear	Unclear	Yes	Yes	Unclear	Yes
**17.** If any of the results of the study were based on “data dredging”, was that made clear?	Yes	Yes	Yes	Yes	Yes	Unclear	Yes	Yes
**18.** Do the analyses adjust for different lengths of follow-up of patients?	Yes	Yes	Unclear	Yes	Yes	No	Yes	Yes
**19.** Were the statistical tests used to assess the main outcomes appropriate?	Yes	Yes	No	No	No	Unclear	Yes	Yes
**20.** Was compliance with the intervention, i.e. appliance wear, reliable?	Yes	Yes	Unclear	Unclear	Unclear	Yes	Yes	Yes
**21.** Was the skeletal maturation staging assessment accurate (valid and reliable)?	Yes	Yes	Unclear	Unclear	Unclear	Unclear	Unclear	No
**22.** Were the main outcomes measures used accurate (valid and reliable)?	Yes	Yes	Yes	Yes	Unclear	Unclear	Yes	Yes
**Internal validity—confounding**								
**23.** Were the patients in different intervention groups recruited from the same population?	No	No	Yes	Yes	No	Unclear	Unclear	Yes
**24.** Were the baseline characteristics comparable?	Yes	Yes	Yes	Yes	Yes	Unclear	Yes	Yes
**25.** Were study subjects in different intervention groups recruited over the same period of time?	No	No	Unclear	No	Unclear	Unclear	Unclear	No
**26.** Was there adequate adjustment for confounding in the analyses from which the main findings were drawn?	No	No	No	No	No	No	No	No
**27.** Were losses of patients to follow-up taken into account?	Yes	Yes	Yes	Yes	Yes	Yes	Yes	Yes
**Power**								
**28.** Prior estimate of sample size	No	No	No	No	No	No	Yes	Yes
**Total**	**20**	**19**	**15**	**16**	**15**	**12**	**24**	**24**

### Sensitivity analysis

Sensitivity analysis is detailed in Table 9. Generally, overall effects proved to be robust enough except for the study with higher risk of bias [40]. Given the results of the sensitivity analysis combined with the overall risk of bias, 1 CCT [40] was excluded from the meta-analyses and GRADE assessment reported below. One study [40] uncovered a relevant effect at the sensitivity analysis. Regarding the pubertal subgroup, the overall (for all studies) total mandibular length and mandibular ramus showed about 0.8 mm difference with the corresponding values without the study with the highest risk of bias assessment [40]. Similarly, clinically relevant effects were seen when removing the same study [40] for the ANB angle and facial divergence. Of note the mandibular incisor proclination also yielded some different estimations between all the studies when a RCT [42] was removed.

**Table 9:** Results of the sensitivity analyses for each of the included parameter according to the pre-pubertal and pubertal subgroups.

Variable	Subgroup	Removed study	Mean Difference [95% CI]
Total mandibular length (mm)	Pre-pubertal	Tulloch et al. [33]	0.71 [0.18, 1.23]
		Baccetti et al. [34]	0.97 [0.60, 1.34]
		Faltin et al. [35]	1.08 [0.71, 1.46]
		Almeida-Pedrin et al. [38]	1.12 [0.74, 1.51]
		Singh et al. [40]	1.05 [0.69, 1.41]
		Perillo et al. [43]	1.17 [0.79, 1.55]
		**All studies**	**1.04 [0.69, 1.4]**
	Pubertal	Baccetti et al. [34]	3.67 [3.17, 4.16]
		Faltin et al. [35]	3.87 [3.40, 4.35]
		Quintão et al. [37]	3.88 [3.40, 4.36]
		Cui et al. [39]	3.91 [3.42, 4.40]
		Singh et al. [40]	2.95 [2.33, 3.57], **a**
		Baysal and Uysal [41], [44]	4.05 [3.55, 4.55]
		Martina et al. [42]	3.91 [3.42, 4.41]
		**All studies**	**3.8 [3.33, 4.26]**
Mandibular ramus height (mm)	Pre-pubertal	Baccetti et al. [34]	-0.06 [-0.65, 0.52]
		Faltin et al. [35]	0.00 [-0.58, 0.58]
		Singh et al. [40]	0.00 [-0.52, 0.52]
		Perillo et al. [43]	0.21 [-0.43, 0.85]
		**All studies**	**0.03 [-0.47, 0.53]**
	Pubertal	Baccetti et al. [34]	2.91 [2.44, 3.39]
		Faltin et al. [35]	2.95 [2.48, 3.41]
		Singh et al. [40]	2.17 [1.50, 2.84], **a**
		Baysal and Uysal [41], [44]	3.07 [2.57, 3.57]
		Martina et al. [42]	3.02 [2.56, 3.49]
		**All studies**	**2.90 [2.45, 3.34]**
Composite mandibular length (mm)	Pre-pubertal	Baccetti et al. [34]	0.94 [0.18, 1.69]
		Faltin et al. [35]	1.21 [0.41, 2.01]
		O'Brien et al. [36]	0.47 [-0.69, 1.63]
		**All studies**	**0.96 [0.25, 1.66]**
	Pubertal	Baccetti et al. [34]	1.77 [1.00, 2.55]
		Faltin et al. [35]	2.36 [1.62, 3.10]
		Baysal and Uysal [41], [44]	2.42 [1.59, 3.25]
		Martina et al. [42]	1.99 [1.12, 2.86]
		**All studies**	**2.14 [1.45, 2.83]**
Mandibular base (mm)	Pre-pubertal	Baccetti et al. [34]	0.77 [0.10, 1.44]
		Faltin et al. [35]	1.00 [0.28, 1.71]
		O'Brien et al. [36]	1.22 [0.07, 2.38]
		**All studies**	**0.93 [0.29, 1.56]**
	Pubertal	Baccetti et al. [34]	1.51 [0.94, 2.07]
		Faltin et al. [35]	1.75 [1.20, 2.30]
		Baysal and Uysal [41], [44]	1.69 [0.96, 2.43]
		Martina et al. [42]	1.58 [0.93, 2.24]
		**All studies**	**1.63 [1.10, 2.16]**
SNA angle (°)	Pre-pubertal	Tulloch et al. [33]	0.02 [-0.30, 0.340]
		Almeida-Pedrin et al. [38]	-0.14 [-0.49, 0.20]
		Singh et al. [40]	-0.02 [-0.29, 0.25]
		Perillo et al. [43]	-0.03 [-0.33, 0.26]
		**All studies**	**-0.04 [-0.30, 0.22]**
	Pubertal	Quintão et al. [37]	-0.42 [-0.73, -0.11]
		Cui et al. [39]	-0.49 [-0.81, -0.18]
		Singh et al. [40]	-0.63 [-0.98, -0.28]
		Baysal and Uysal [41], [44]	-0.16 [-0.59, 0.27]
		**All studies**	**-0.45 [-0.75, -0.16]**
SNB angle (°)	Pre-pubertal	Tulloch et al. [33]	0.49 [0.19, 0.79]
		Almeida-Pedrin et al. [38]	0.43 [0.17, 0.69]
		Singh et al. [40]	0.58 [0.35, 0.81]
		Perillo et al. [43]	0.70 [0.45, 0.95]
		**All studies**	**0.56 [0.33, 0.78]**
	Pubertal	Quintão et al. [37]	1.92 [1.57, 2.26]
		Cui et al. [39]	2.00 [1.64, 2.37]
		Singh et al. [40]	1.00 [0.60, 1.39], **a**
		Baysal and Uysal [41], [44]	2.09 [1.69, 2.50]
		**All studies**	**1.77 [1.44, 2.09]**
ANB angle (°)	Pre-pubertal	Tulloch et al. [33]	-0.71 [-1.01, -0.42]
		Almeida-Pedrin et al. [38]	-0.74 [-1.00, -0.47]
		Singh et al. [40]	-0.73 [-0.95, -0.51]
		Perillo et al. [43]	-0.76 [-0.99, -0.52]
		**All studies**	**-0.73 [-0.95, -0.52]**
	Pubertal	Quintão et al. [37]	-2.10 [-2.42, -1.77]
		Cui et al. [39]	-2.14 [-2.46, -1.82]
		Singh et al. [40]	-1.55 [-1.89, -1.22]
		Baysal and Uysal [41], [44]	-1.94 [-2.29, -1.58]
		**All studies**	**-1.94 [-2.23, -1.65]**
Maxillary base (mm)	Pre-pubertal	Baccetti et al. [34]	-0.72 [-1.11, -0.34]
		Faltin et al. [35]	-0.59 [-0.98, -0.19]
		O'Brien et al. [36]	-0.47 [-1.10, 0.16]
		**All studies**	**-0.63 [-0.98, -0.27]**
	Pubertal	Baccetti et al. [34]	-0.46 [-0.84, -0.08]
		Faltin et al. [35]	-0.57 [-0.95, -0.20]
		Baysal and Uysal [41], [44]	-0.33 [-0.85, 0.19]
		Martina et al. [42]	-0.52 [-0.89, -0.15]
		**All studies**	**-0.49 [-0.84, -0.15]**
Facial divergence (°)	Pre-pubertal	Baccetti et al. [34]	0.04 [-0.30, 0.37]
		Faltin et al. [35]	0.03 [-0.31, 0.37]
		Almeida-Pedrin et al. [38]	-0.02 [-0.40, 0.36]
		Singh et al. [40]	0.16 [-0.17, 0.49]
		Perillo et al. [43]	0.38 [-0.02, 0.79]
		**All studies**	**0.11 [-0.21, 0.42]**
	Pubertal	Baccetti et al. [34]	1.54 [1.13, 1.94]
		Faltin et al. [35]	1.58 [1.17, 1.99]
		Cui et al. [39]	1.52 [1.12, 1.93]
		Singh et al. [40]	0.80 [0.33, 1.26], **a**
		Baysal and Uysal [41], [44]	1.66 [1.25, 2.06]
		Martina et al. [42]	1.53 [1.13, 1.93]
		**All studies**	**1.46 [1.09, 1.84]**
Mandibular incisors proclination (°)	Pre-pubertal	Almeida-Pedrin et al. [38]	1.64 [0.02, 3.27]
		Singh et al. [40]	1.37 [0.38, 2.36]
		Perillo et al. [43]	1.17 [0.02, 2.32]
		**All studies**	**1.35 [0.39, 2.31]**
	Pubertal	Cui et al. [39]	0.94 [-0.20, 2.08]
		Singh et al. [40]	0.72 [-0.32, 1.77]
		Baysal and Uysal [41], [44]	1.01 [-0.28, 2.30]
		Martina et al. [42]	0.17 [-0.95, 1.29], **a**
		**All studies**	**0.69 [-0.29, 1.68]**

Note of judgments:

**a**, value with clinical relevant difference as compared to the corresponding overall (all studies) mean.

### Risk of bias among studies

**Fig 2:**
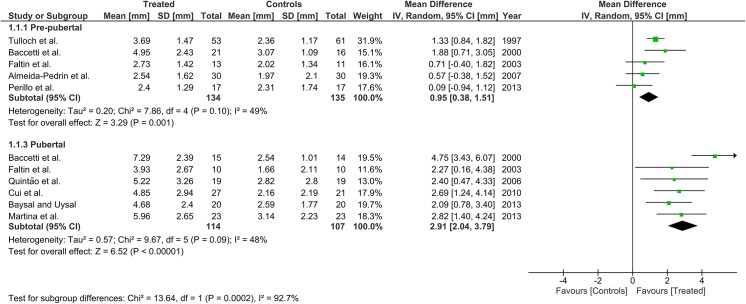
Forest plots for the annualised changes in total mandibular length according to the pre-pubertal and pubertal subgroups.

**Fig 3:**
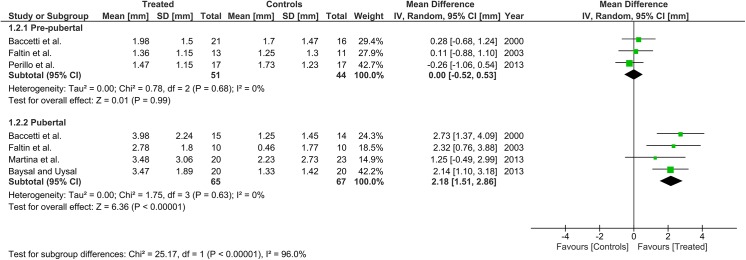
Forest plots for the annualised changes in mandibular ramus height according to the pre-pubertal and pubertal subgroups.

**Fig 4:**
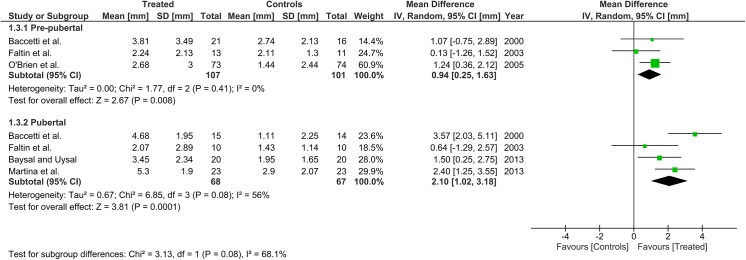
Forest plots for the annualised changes in composite mandibular length (Pancherz analysis) according to the pre-pubertal and pubertal subgroups.

**Fig 5:**
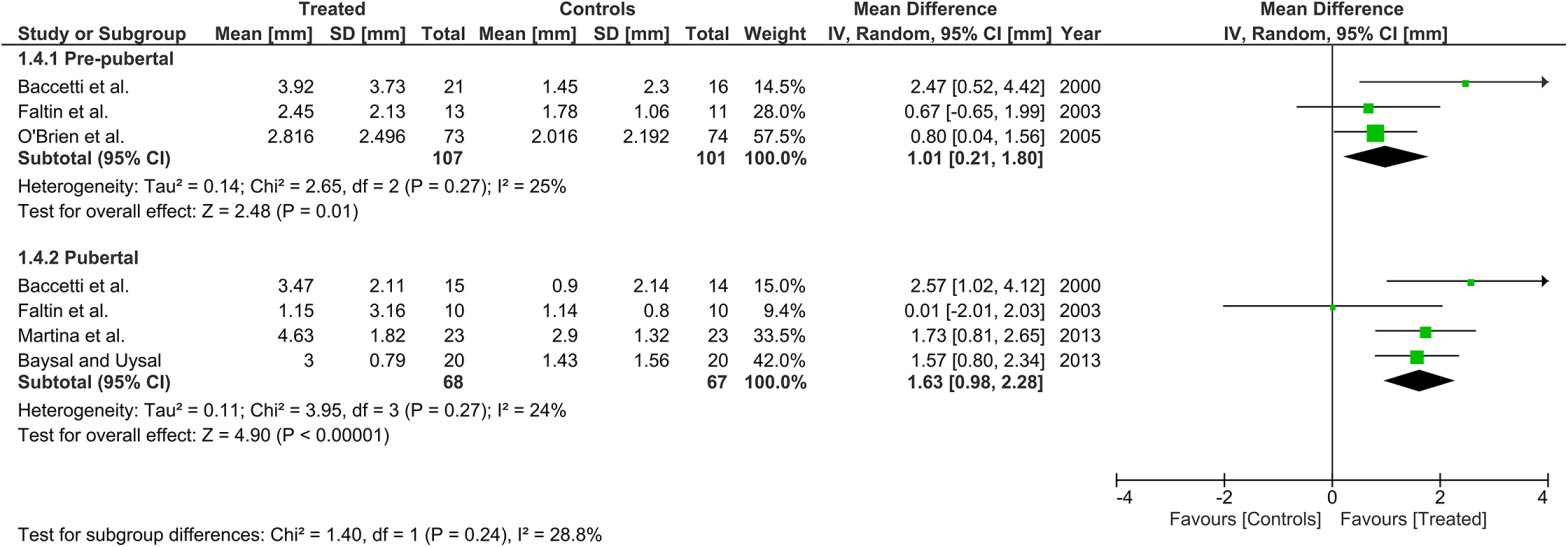
Forest plots for the annualised changes in mandibular base (Pancherz analysis) according to the pre-pubertal and pubertal subgroups.

**Fig 6:**
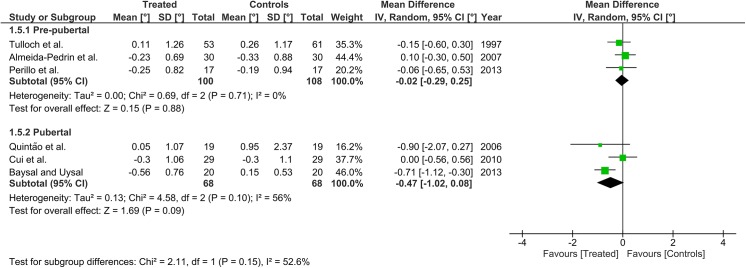
Forest plots for the annualised changes in SNA angle according to the pre-pubertal and pubertal subgroups.

**Fig 7:**
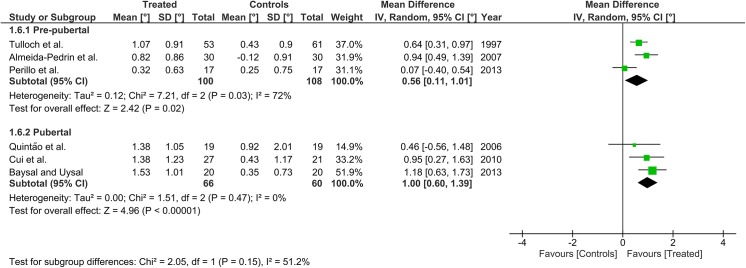
Forest plots for the annualised changes in SNB angle according to the pre-pubertal and pubertal subgroups.

**Fig 8:**
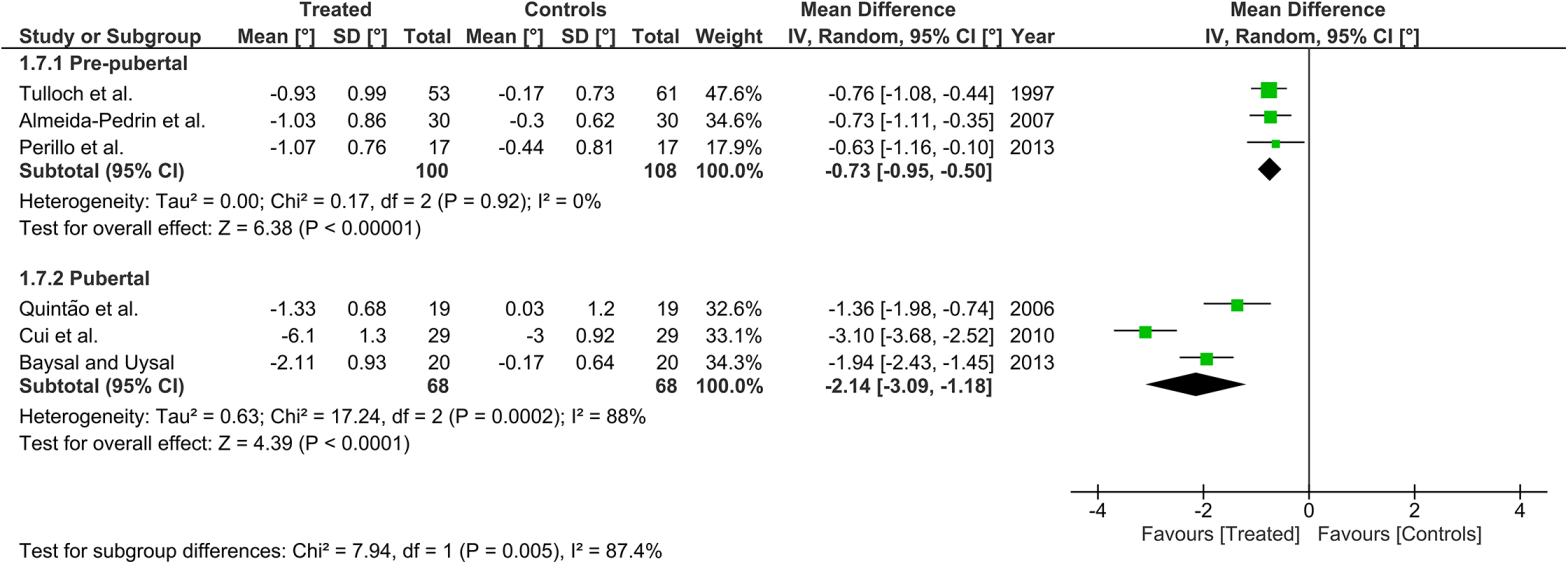
Forest plots for the annualised changes in ANB angle according to the pre-pubertal and pubertal subgroups.

**Fig 9:**
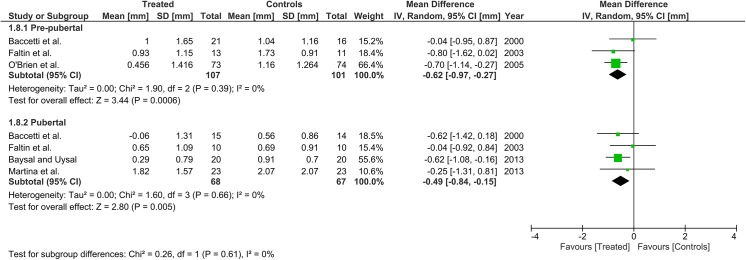
Forest plots for the annualised changes in maxillary base (Pancherz analysis) according to the pre-pubertal and pubertal subgroups.

**Fig 10:**
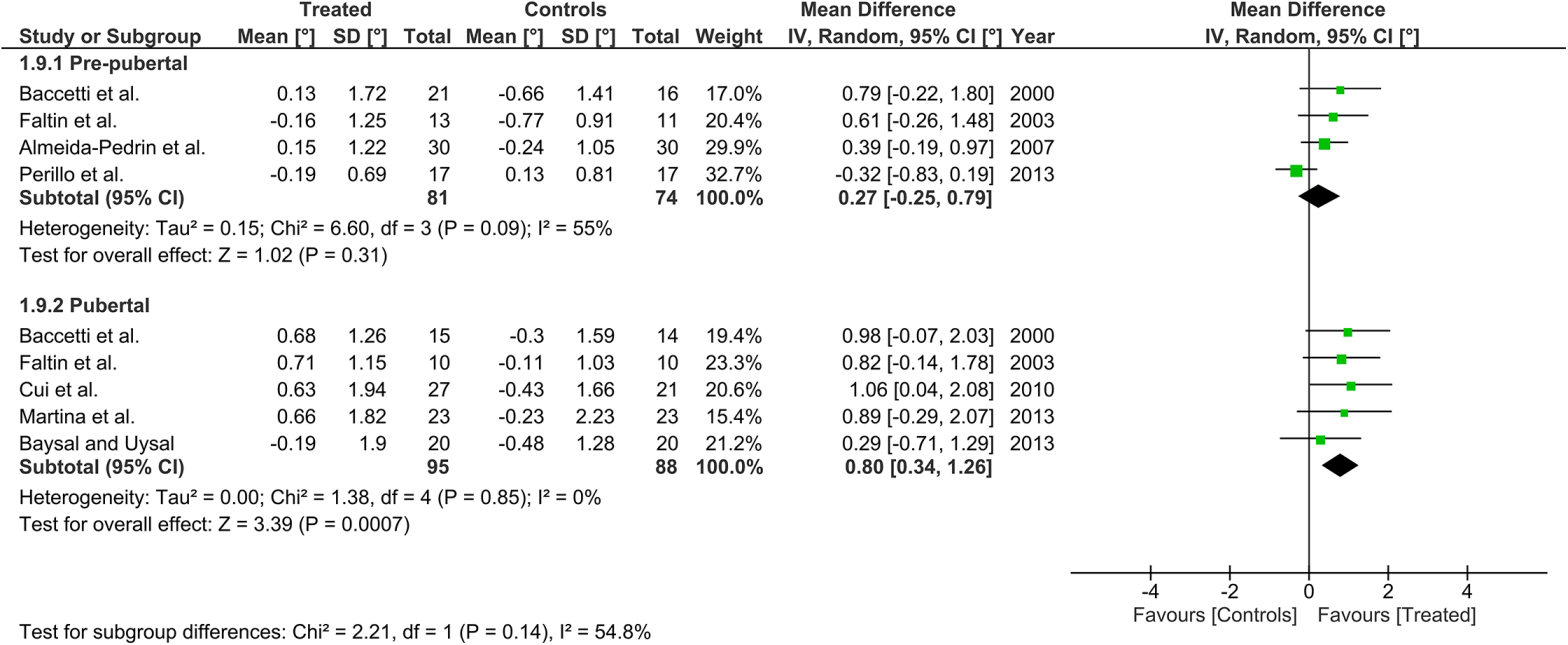
Forest plots for the annualised changes in facial divergence according to the pre-pubertal and pubertal subgroups.

**Fig 11:**
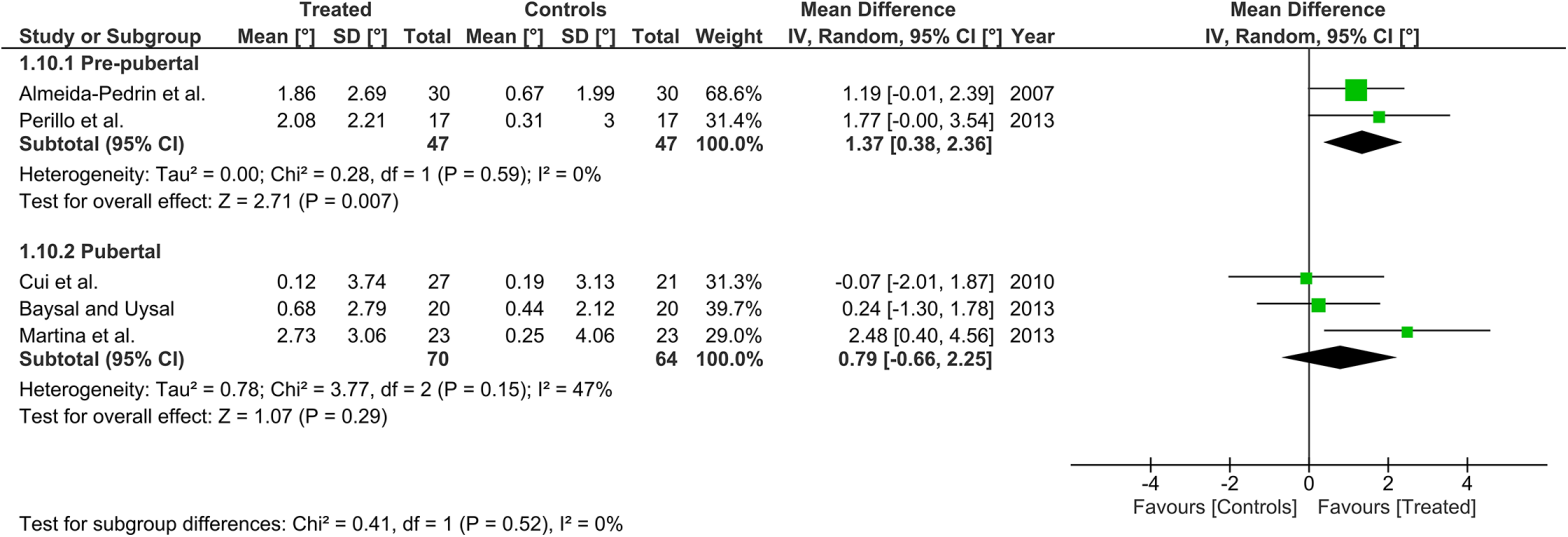
Forest plots for the annualised changes in mandibular incisor proclination according to the pre-pubertal and pubertal subgroups.

Heterogeneity at the subgroup level was generally low, with I^2^ values between 0% and 56% for all the primary outcomes (Figs 2–5). On the contrary, substantial heterogeneity was seen for the SNA, SNB, ANB angles with I^2^ values up to 88% (ANB angle, pubertal subgroup) as shown in Figs 6–8. The maxillary base (Pancherz Analysis) and facial divergence showed no or acceptable heterogeneity with I^2^ values equal to 0 in both subgroups (Fig 9) or not exceeding 55% (Fig 10), respectively. Finally, lower incisor proclination also showed acceptable heterogeneity with I^2^ values not exceeding 47% in both subgroups (Fig 11). Results on the publication bias analyses are shown in Table 10. Generally non-significant p values were seen for all the parameters in both subgroups. Exception were seen for the SNB and ANB angles that yielded a significant publication bias according to the Egger test in the pubertal subgroup (p = 0.020 and p = 0.056, respectively), for the ANB for the pre-pubertal subgroup (p = 0.055), and for the facial divergence for the pre-pubertal subgroup (p = 0.089).

**Table 10:** The p values of the Egger regression intercept test and Begg and Mazumdar rank correlation test on the on the publication bias analyses for each of the included parameter according to the pre-pubertal and pubertal subgroups.

Variable	Subgroup	Test	
		****Egger****	****Begg and Mazumdar****
Total mandibular length	Pre-pubertal	0.467	1.000
	Pubertal	0.551	0.452
Mandibular ramus height	Pre-pubertal	0.213	1.000
	Pubertal	0.717	0.734
Composite mandibular length	Pre-pubertal	0.646	1.000
	Pubertal	0.752	0.734
Mandibular base	Pre-pubertal	0.472	0.296
	Pubertal	0.750	1.000
SNA	Pre-pubertal	0.706	1.000
	Pubertal	0.798	1.000
SNB	Pre-pubertal	0.816	1.000
	Pubertal	0.020**,** **a**	0.296
ANB	Pre-pubertal	0.055, **a**	0.296
	Pubertal	0.056, **a**	0.296
Maxillary base	Pre-pubertal	0.296	0.603
	Pubertal	0.278	0.308
Facial divergence	Pre-pubertal	0.193	0.089, **a**
	Pubertal	0.643	0.806
Mandibular incisor proclination	Pre-pubertal	—	—
	Pubertal	0.628	1.000

Publication bias analysis does not include the study by Singh *et al*. [40] excluded according to the risk of bias and sensitivity analyses. Further notes:

**a**, significant p value denoting publication bias; —, p value not derivable since only 2 studies were included.

### Meta-analysis for the primary outcomes

The cephalometric measurements used in each study and pooled herein for the meta-analysis are reported in Table 11. Detailed results for the meta-analysis for the primary outcomes are shown in Figs 2–5. Overall effects are expressed as mean (95% confidence interval) with 90% prediction intervals summarised in Table 12. For the total mandibular length, no study made use of the Articulare as the endpoint. The overall annualised changes were 0.95 mm (0.38, 1.51) and 2.91 mm (2.04, 3.79) in the pre-pubertal and pubertal subgroups, respectively. The difference between the subgroups was significant at p<0.01 (Fig 2). The prediction intervals of the annualised changes ranged from -0.30 to 2.20 mm and from 1.04 to 4.78 mm in the pre-pubertal and pubertal subgroups, respectively. Regarding the mandibular ramus height, the overall annualised change in pre-pubertal patients was 0.00 mm (-0.52, 0.53). While in pubertal patients, the overall annualised change was 2.18 mm (1.51, 2.86). The difference between the subgroups was significant at p<0.01 (Fig 3). The prediction intervals of the annualised changes ranged from -1.69 to 1.69 mm and from 1.17 to 3.19 mm in the pre-pubertal and pubertal subgroups, respectively. For the composite mandibular length, the overall annualised change in pre-pubertal patients was 0.94 mm (0.25, 1.63), while in pubertal patients, the overall annualised change was 2.10 mm (1.02, 3.18). The difference between the subgroups was not significant even though the p value was close to significance at 0.08 (Fig 4). The prediction intervals of the annualised changes ranged from -1.28 to 3.16 mm and from -0.78 to 4.98 mm in the pre-pubertal and pubertal subgroups, respectively. Regarding the mandibular base (Pancherz Analysis), the overall annualised change in pre-pubertal patients was 1.01 mm (0.21, 1.80), while in pubertal patients, the overall annualised change was 1.63 mm (0.98, 2.28), without significant differences between subgroups (p = 0.24; Fig 3). The prediction intervals of the annualised changes ranged from -2.47 to 4.49 mm and from 0.26 to 3.00 mm in the pre-pubertal and pubertal subgroups, respectively.

**Table 11:** The cephalometric measurements that were pooled for the meta-analyses.

Study	Total mandibular length (mm)	Mandibular ramus height (mm)	Composite mandibular length (Pancherz analysis, mm)	Mandibular base (Pancherz analysis, mm)	Maxillary base (Pancherz analysis, mm)	Facial divergence (°)	Mandibular incisors proclination (°)
Tulloch et al. [33]	Md unit length, a	NA	NA	NA	NA	NA	NA
Baccetti et al. [34]	Co-Pg	Co-Go	Pg/OLp + Co/OLp	Pg/OLp	A point/OLp	ml/FMN-T line	NA
Faltin et al. [35]	Co-Pg	Co-Go	Pg/OLp + Co/OLp	Pg/OLp	A point/OLp	ml/FMN-T line	NA
O'Brien et al. [36]	NA	NA	Pg/OLp + Co/OLp	Pg/OLp	A point/OLp	NA	NA
Quintão et al. [37]	Co-Gn	NA	NA	NA	NA	NA	NA
Almeida-Pedrin et al. [38]	Co-Gn	NA	NA	NA	NA	SN.GoGn	IMPA
Cui et al. [39]	Co-Gn	NA	NA	NA	NA	NA	L1-MP
Singh et al. [40]	Cd-Gn	Cd-Go	NA	NA	NA	FMA	LI-MnP
Baysal and Uysal [41], [44]	Co-Gn	Co-Go	Pg/OLp + Co/OLp	Pg/OLp	A point/OLp	SN-GoGn	IMPA
Martina et al. [42]	Co-Pg	Co-Go	Pg/OLp + Co/OLp	Pg/OLp	Ss point/OLp	SN-MP	IMPA
Perillo et al. [43]	Co-Gn	Co-Go, **b**	NA	NA	NA	FH-MP	L1/MP

**Co** or **Cd,** Condylion; **Go**, Gonion; **Gn**, Gnathion; **Pg**, Pogonion; **Md**, mandibular; **MP** or **MnP**, mandibular plane; **ml**, mandibular line; **FMN**, fronto-maxillo-nasal suture; **FH**, Frankfurt horizontal; **L1 or LI or 1-**, mandibular incisor axis; **IMPA**, lower incisor mandibular plane angle; **NA**, not available. Pancherz analysis according to a previous report [27]. Further notes:

**a**, reference points not provided

**b**, data provided by the Authors.

**Table 12:** The 95% Prediction intervals for each of the included parameter according to the pre-pubertal and pubertal subgroups.

Variable	Subgroup (n of studies)	Point estimate [90% PI)
**Primary outcome**		
Total mandibular length (mm)	Pre-pubertal (5)	0.95 [-0.30, 2.20]
	Pubertal (6)	2.91 [1.04, 4.78]
Mandibular ramus height (mm)	Pre-pubertal (3)	0.00 [-1.69, 1.69]
	Pubertal (4)	2.18 [1.17, 3.19]
Composite mandibular length (mm)	Pre-pubertal (3)	0.94 [-1.28, 3.16]
	Pubertal (4)	2.10 [-0.78, 4.98]
Mandibular base (mm)	Pre-pubertal (3)	1.01 [-2.47, 4.49]
	Pubertal (4)	1.63 [0.26, 3.00]
**Secondary outcome**		
SNA angle (°)	Pre-pubertal (3)	-0.02 [-0.89, 0.85]
	Pubertal (3)	-0.47 [-3.35, 2.41]
SNB angle (°)	Pre-pubertal (3)	0.56 [-2.06, 3.18]
	Pubertal (3)	1.00 [-0.27, 2.27]
ANB angle (°)	Pre-pubertal (3)	-0.73 [-1.45, -0.01]
	Pubertal (3)	-2.14 [-8.02, 3.74]
Maxillary base (mm)	Pre-pubertal (3)	-0.69 [-1.75, 0.51]
	Pubertal (4)	-0.49 [-1.00, 0.02]
Facial divergence (°)	Pre-pubertal (4)	0.27 [-1.10, 1.64]
	Pubertal (5)	0.80 [0.25, 1.35]
Mandibular incisors proclination (°)	Pre-pubertal (2)	1.37 [––,––]
	Pubertal (3)	0.79 [-6.49, 8.07]

**PI**, prediction intervals, —, prediction interval not derivable since only 2 studies were included. Refer to Figs 2–11 for studies included in each subgroup analysis.

### Meta-analysis for the secondary outcomes

The cephalometric measurements used in each study, and pooled herein for the meta-analysis are reported in Table 11. Detailed results for the meta-analysis are shown in Figs 6–11 for the secondary outcomes with 90% prediction intervals summarised in Table 12. Overall effects are expressed as mean (95% confidence interval). For the SNA angle, the overall annualised change in pre-pubertal patients was -0.02° (-0.29, 0.25). While in pubertal patients, the overall annualised change was -0.05° (-1.02, 0.08), but the difference between the two subgroups was not significant at p = 0.15, and the I^2^ values were 0% and 56% for the pre-pubertal and pubertal subgroups, respectively (Fig 6). The prediction intervals of the annualised changes ranged from -0.89° to 0.85° and from -3.35° to 2.41° in the pre-pubertal and pubertal subgroups, respectively. Regarding the SNB angle, the overall annualised change in pre-pubertal patients was 0.56° (0.11, 1.01) and of 1.00° (0.60, 1.39) in pubertal patients, with no significant (p = 0.15) differences between the subgroups, and the I^2^ values were 72% and 0% for the pre-pubertal and pubertal subgroups, respectively (Fig 7). The prediction intervals of the annualised changes ranged from -2.06° to 3.18° and from -0.27° to 2.27° in the pre-pubertal and pubertal subgroups, respectively. For the ANB angle, the overall annualised change in pre-pubertal patients was -0.73° (-0.95, -0.50) while, in pubertal patients, the overall annualised change was -2.14° (-3.09, -1.18). The difference between the subgroups was significant at p<0.01, and the I^2^ values were 0% and 88% for the pre-pubertal and pubertal subgroups, respectively (Fig 8). The prediction intervals of the annualised changes ranged from -1.45° to -0.01° and from -8.02° to 3.74° in the pre-pubertal and pubertal subgroups, respectively. Regarding the Maxillary base (Pancherz Analysis), the overall annualised change in pre-pubertal patients was -0.62 mm (-0.97, -0.27) and -0.49 mm (-0.84, -0.15) in pubertal patients. The difference between the subgroups was not significant at p = 0.66, and the I^2^ values were 0% for both the subgroups (Fig 9). The prediction intervals of the annualised changes ranged from –1.75 to 0.51 mm and from -1.00 to 0.02 mm in the pre-pubertal and pubertal subgroups, respectively. For the facial divergence, the overall annualised change in pre-pubertal patients was 0.27° (-0.25, 0.79), while in pubertal patients, the overall annualised change was 0.80° (0.34, 1.26). The difference between the subgroups was not significant at p = 0.14, and the I^2^ values were 55% and 0% for the pre-pubertal and pubertal subgroups, respectively (Fig 10). The prediction intervals of the annualised changes ranged from -1.10° to 1.64° and from -0.25° to 1.35° in the pre-pubertal and pubertal subgroups, respectively. Finally, for the mandibular incisor proclination, the overall annualised change in pre-pubertal patients was 1.37° (0.38, 2.36) and 0.79° (-0.66, 2.25) in pubertal patients. The difference between the subgroups was not significant at p = 0.52, and the I^2^ values were 0% and 47% for the pre-pubertal and pubertal subgroups, respectively (Fig 11). The prediction intervals of the annualised changes was not derivable for the pre-pubertal patients, while for the pubertal patients ranged from -6.49° to 8.07°.

### GRADE Assessment

The GRADE assessment for each of the primary outcome with detailed information is shown in Table 13. For the pre-pubertal patients, the quality of evidence was low for all the outcomes. For the pubertal patients, the overall quality was between low (composite mandibular length) to moderate (for all the other outcomes). Reasons for downgrading were related to the items ‘risk of bias’ (use of CCT, historical controls, and other bias as stated above) and ‘imprecision’ (according to the heterogeneity seen) and for inclusion of small studies. No downgrading was assessed for the inconsistency, indirectness or publication bias (according to the results of the analyses reported above). Finally, upgrading mainly responsible for the greater quality seen in the pubertal subgroup as compared to the pre-pubertal one was due to the dimension of the treatment effect for total mandibular length, mandibular ramus height and mandibular base that reached a ‘large effect’.

**Table 13:** The GRADE assessment for each of the primary outcomes, according to the pre-pubertal and pubertal subgroups.

Outcomes	No of Participants/ studies/ Follow up	Quality of the evidence (GRADE)	Anticipated absolute effects	
			Risk with Control	Risk difference with functional treatment (95% CI)
**Pre-pubertal subjects**				
Total mandibular length	269/ 5 studies/ 1.0–1.8 years	**LOW** a **,** b - due to risk of bias, imprecision	The mean total mandibular length ranged across control groups from **2.02 to 3.07 mm**	The mean total mandibular length in the intervention groups was **0.95 higher** (0.38 to 1.51 higher)
Mandibular ramus height	135/ 3 studies/ 1.0–1.8 years	**LOW c,d—**due to risk of bias, imprecision	The mean mandibular ramus height ranged across control groups from **1.25 to 1.73 mm**	The mean mandibular ramus height in the intervention groups was **0.00 higher** (0.52 lower to 0.53 higher)
Composite mandibular length (Pancherz analysis)	208/ 3 studies/ 1.0–1.8 years	**LOW a,d—**due to risk of bias, imprecision	The mean composite mandibular length (Pancherz analysis) ranged across control groups from **1.44 to 2.74 mm**	The mean composite mandibular length (Pancherz analysis) in the intervention groups was **0.94 higher** (0.25 to 1.63 higher)
Mandibular base (Pancherz analysis)	208/ 3 studies/ 1.0–1.8 years	**LOW a,b** - due to risk of bias, imprecision	The mean mandibular base (Pancherz analysis) ranged across control groups from **1.45 to 2.02 mm**	The mean mandibular base (Pancherz analysis) in the intervention groups was **1.01 higher** (0.21 to 1.8 higher)
**Pubertal subjects**				
Total mandibular length	221/ 6 studies/ 1.0–2.3 years	**MODERATE b,c,e** - due to risk of bias, imprecision, large effect	The mean total mandibular length ranged across control groups from **1.66 to 3.14 mm**	The mean total mandibular length in the intervention groups was **2.91 higher** (2.04 to 3.79 higher)
Mandibular ramus height	132/ 4 studies/ 1.0–2.3 years	**MODERATE a,d,^f^** - due to risk of bias, imprecision, large effect	The mean mandibular ramus height ranged across control groups from **0.46 to 2.23 mm**	The mean mandibular ramus height in the intervention groups was **2.18 higher** (1.51 to 2.86 higher)
Composite mandibular length (Pancherz analysis)	135/ 4 studies/ 1.0–2.3 years	**LOW a,b** - due to risk of bias, imprecision	The mean composite mandibular length (Pancherz analysis) ranged across control groups from **1.11 to 1.95 mm**	The mean composite mandibular length (Pancherz analysis) in the intervention groups was **2.10 higher** (1.02 to 3.18 higher)
Mandibular base (Pancherz analysis)	135/ 4 studies/ 1.0–2.3 years	**MODERATE a,b,g**—due to risk of bias, imprecision, large effect	The mean mandibular base (Pancherz analysis) ranged across control groups from **0.90 to 1.43 mm**	The mean mandibular base (Pancherz analysis) in the intervention groups was **1.63 higher** (0.98 to 2.28 higher)

**CI**, confidence interval. Notes on the GRADE assessment:

**a**, only 1 randomised study, historical controls, other less relevant biases

**b**, different treatment durations/observation, modest heterogeneity, 1 small study

**c**, no randomised study, historical controls, other less relevant biases

**c**, different treatment durations/observation, 1 small study

**e**, additional mandibular elongation above 2.5 mm/year

**f**, additional mandibular elongation above 2.0 mm/year

## Discussion

The present review allowed the comparison of the effects of functional treatment of skeletal Class II malocclusion by removable appliances between pre-pubertal and pubertal patients. Study designs and main results at the skeletal, dentoalveolar and soft tissue levels were reviewed. Moreover, cephalometric parameters, mainly regarding mandibular growth, were meta-analysed. Overall, taking into account relevant individual variations, the present results demonstrate clinically relevant skeletal effects in terms of additional mandibular growth only if treatment is performed during the pubertal growth phase.

In spite of the large number of studies initially retrieved (Fig 1), most of them analysed in full-text were excluded because they did not consider a reliable indicator of skeletal maturity, or because they lacked of an untreated Class II control group (Table 3). Interestingly, a relevant RCT on pubertal subjects [42] was missed in one of the most recent meta-analyses [6].

Even though different treatment modalities were followed in the included studies (and after removal of a low quality investigation [40], heterogeneity among the studies was acceptable with I^2^ mostly below 50% for the primary outcomes and some secondary outcomes (Figs 2–5) with consistent results. On the contrary, SNA, SNB and ANB angles showed substantial heterogeneity. Of note, heterogeneity seen herein at the subgroup level for the main outcomes was generally below those reported in other similar investigations [12] where the growth phase was not considered as a clustering factor. Therefore, the different growth phase may explain part of the heterogeneity (and apparent inconsistency of the results) previously reported.

Herein, clinically relevant effects in terms of additional mandibular elongation was see for the pubertal patients of 2.91 mm/year (Fig 2). Similar clinically relevant results were seen herein for the additional increment of the mandibular ramus height (Fig 3). However, different removable appliances may have different *modus operandi* requiring differential treatment duration. A previous meta-analysis [4] reported no significant effects of functional treatment in Class II patients. This meta-analysis used standardised mean differences (obtained merging several parameters) for the estimation of the overall effects. However, while standardised mean differences may give an indication of the variability among individuals, they do not describe the magnitude of the effect. Further meta-analyses reported some skeletal effects for functional treatment of Class II malocclusion by the use of the Functional Regulator-2 [12] and Twin-Block [7], even though the Authors were not conclusive in terms of treatment efficiency. On the contrary, the results of the present study on pubertal patients may be compared with those from a recent meta-analysis [13] on fixed functional appliance where the mean additional mandibular (total length) growth as compared to matched untreated subjects was about 2 mm. Even though this previous meta-analysis did not report annualised changes it might be hypothesised that, irrespective of the fixed or removable appliance used, skeletal effects are dependent on the growth phase (pubertal) during which treatment is performed.

Of note, a noteworthy individual variation in terms of treatment responsiveness was also seen in pubertal patients particularly for the annualised total mandibular length increment (prediction interval from 1.04 to 4.78 mm, Table 12). In this regard, individual variations has been previously reported in pubertal Class II patients treated by functional appliances with the condylar angle as one of the prognostic feature [10]. Interestingly, none of the included studies herein has classified patients according to this prognostic feature (Table 5). However, the present meta-analysis may not discriminate whether such individual variation in treatment effects was due to the different treatment protocols, patient’s compliance or biological individual responsiveness. In spite of these aspects, the present results would be consistent with previous findings reporting insulin growth factor 1 among the key factors promoting chondrogenesis of the condylar cartilage [45], the serum levels of which would be to be significantly greater in the pubertal as compared to pre-pubertal subjects, as determined though the CVM method [46], [47].

While a relevant ‘headgear’ effect has been reported for the fixed functional appliances used during the pubertal growth phase [13], herein, irrespective of the growth phase of the patients, a limited maxillary growth restrain (Figs 6 and 9, Table 12) was seen. Taking also into account previous findings [7], it may be hypothesised that removable and fixed functional appliances have different effects on maxillary bone.

An increase in facial divergence was not seen herein (Fig 10, Table 12) while, a slightly greater (although not significant) mandibular incisors proclination was seen for pre-pubertal patients (Fig 11, Table 12). However, this proclination appears to be of limited clinical relevance in either pre-pubertal or pubertal patients. On the contrary, increase in both these parameters have been reported earlier for the Twin-Block treatment [7]. The individual management of the dentition, i.e. extrusion of mandibular teeth, during treatment may explain at least part of this apparent inconsistency.

### Limitations of the review

The current investigation on the effects of functional treatment of Class II malocclusion is inherently hampered by some factors. In spite of the use of annualised changes, observational terms may include not only the effective functional treatment, but also variable periods of time of retention, or of further management of the dentition. Therefore, skeletal changes might occur not uniformly during the entire observational term skewing the analysis of treatment outcomes [5]. The studies included were mostly CCTs, and in 5 studies treated groups followed a retrospective enrolment of the treated group [34], [35], [39], [40], [43] (Table 5). Hardly to be avoided, heterogeneity of the selected studies was mainly seen in the treatment duration, type of appliance used (even though they all share the mechanism of forward posturing of the mandible), or severity of malocclusion (Table 5). Moreover, 2 studies [33], [36] used overjet as the only diagnostic criterion for Class II malocclusion, even though in 1 study [33], likely most of the patients had a skeletal Class II malocclusion according to mean ±SD of ANB angle of ∼6.3° ±2.0°. One study [40] was judged to be affected by a significant risk of bias (Table 8) and had to be excluded from the meta-analysis. Some of the included studies had small sample sizes [35], [40], and in 2 studies [37], [39] cephalometric magnification was not declared or retrieved (even though linear measurement used herein were not reported in those investigations, the rest of the data were set at 0% magnification). Moreover, similar skeletal outcomes were defined slight differently at the cephalometric recording (see above and Table 11). Finally, an analysis of the potential responsiveness to treatment according to gender or other prognostic factors was not feasible, and this review has focused on short-term effects.

The GRADE quality of evidence assessment was moderate for several main outcomes (Table 13) mainly due to the large effect assigned to these outcomes according to the re-establishment of normal growth in Class II patients [32]. However, studies with an improved level of quality are necessary, with regard to prospective enrolment, full description of Class II features, adequate statistical analysis, and other related information. Even RCTs should rely on skeletal assessment of Class II malocclusion instead of using the overjet, which is more indicative of prominent upper frontal teeth and not always associated with a genuine Class II skeletal pattern [21].

### Clinical implications

Within the limitations and heterogeneity of the included studies it appears that, in spite of the specific type of appliance used and the protocol followed, functional treatment with removable appliances would be valid in correcting skeletal Class II malocclusion. However, the effects behind the correction would be related to treatment timing. Skeletal corrections, including mainly mandibular elongation with minimal or no maxillary growth restrain, may be achieved if treatment is performed during the pubertal rather than pre-pubertal growth phase. All the radiographical methods used in the included studies both based on the HWM [33], [37], [39], [41], [43], [44] and CVM method [34–36], [38], [40], [42] methods that have been shown to be related to the mandibular growth spurt and stature height [48], [49], [50]. Moreover, the CVM method has showed to be repeatable to a satisfactorily level when executed by trained operators [51]. Finally, a simplified third finger maturation (derived from full HWM) and CVM methods have showed a good degree of correlation and diagnostic agreement, suggesting a combined use according to the available radiographical record [52]. This would be particularly useful when skeletal maturations has to be followed longitudinally in pre-pubertal patients until the beginning of the pubertal growth phase. However, a pure skeletal effect would not be expected even during puberty, as some dentoalveolar effects are also present, even though, mandibular incisor proclination consequent to functional treatment would be limited with minimal clinical implications, especially for pubertal patients. Similarly, an increase of facial divergence was very minimal or absent in both pre-pubertal and pubertal patients. Even though further evidence is needed, the use of a reliable indicator of skeletal maturity either HWM or CVM may be recommended in routine clinical practice to make efforts to perform treatment during the pubertal growth phase.

## Conclusions

Taking into account the still limited quality of the reported studies, and their heterogeneity in terms of study designs, treatment protocols and appliances used, the following conclusion may be drawn:

- Functional treatment by removable appliances may be effective in correcting Class II malocclusion with relevant skeletal effects if performed during the pubertal growth phase. Skeletal effects of functional treatment were seen at the mandibular level and consist mainly in mandibular elongation and increase in ramus height, although dentoalveolar effects were detected even in pubertal patients.
- However, both the increases in total mandibular length and in ramus height showed a noteworthy individual variation to treatment responsiveness in pubertal patients.
- Irrespective of the growth phase, no or very minimal effects were seen in terms of maxillary growth restrain or increase in facial divergence
- Further high quality RCTs with proper inclusion criteria for skeletal Class II malocclusion are needed to fully elucidate the role of growth phase in the efficiency of functional treatment with removable appliances

## Supporting Information

S1 PRISMA Checklist (DOC)

S1 Table Main data set underlying the meta-analysis as RevMan file format. (RM5)
